# ﻿New segregates from the Neotropical genus *Stryphnodendron* (Leguminosae, Caesalpinioideae, mimosoid clade)

**DOI:** 10.3897/phytokeys.205.82220

**Published:** 2022-08-22

**Authors:** Alexandre G. de Lima, Juliana de Paula-Souza, Jens J. Ringelberg, Marcelo F. Simon, Luciano P. de Queiroz, Leonardo M. Borges, Vidal de F. Mansano, Vinicius C. Souza, Viviane R. Scalon

**Affiliations:** 1 Escola Nacional de Botânica Tropical, Instituto de Pesquisas do Jardim Botânico do Rio de Janeiro, Rua Pacheco Leão 2040, 22460-030, Rio de Janeiro/RJ, Brazil Instituto de Pesquisas do Jardim Botânico do Rio de Janeiro Rio de Janeiro Brazil; 2 Department of Biological and Environmental Sciences, University of Gothenburg, Gothenburg, Sweden University of Gothenburg Gothenburg Sweden; 3 Universidade Federal de Santa Catarina, Departamento de Botânica/ CCB. Rua Eng. Agronômico Andrei Cristian Ferreira 216, 88040-535, Florianópolis/SC, Brazil Universidade Federal de Santa Catarina Florianópolis Brazil; 4 Department of Systematic and Evolutionary Botany, University of Zurich, Zollikerstrasse 107, CH-8008, Zurich, Switzerland University of Zurich Zurich Switzerland; 5 Empresa Brasileira de Pesquisa Agopecuária (Embrapa) Recursos Genéticos e Biotecnologia, Parque Estação Biológica, Caixa Postal 02372, 70770-917, Brasília/DF, Brazil Empresa Brasileira de Pesquisa Agopecuária Brasília Brazil; 6 Universidade Estadual de Feira de Santana, Depto. de Ciências Biológicas. Av. Transnordestina s.n., Novo Horizonte, 44036-900, Feira de Santana/BA, Brazil Universidade Estadual de Feira de Santana Feira de Santana Brazil; 7 Universidade Federal de São Carlos, Departamento de Botânica, Rodovia Washington Luís, Km 235, 13565-905, São Carlos/SP, Brazil Universidade Federal de São Carlos São Carlos Brazil; 8 Universidade de São Paulo, Escola Superior de Agricultura “Luiz de Queiroz”, Av. Pádua Dias 11, C.P. 09, 13418-900, Piracicaba/SP, Brazil Universidade de São Paulo Piracicaba Brazil; 9 Universidade Federal de Ouro Preto, Herbário OUPR. Campus Morro do Cruzeiro s.n., 35400-000, Ouro Preto/MG, Brazil Universidade Federal de Ouro Preto Ouro Preto Brazil

**Keywords:** Non-monophyly is a prominent issue in mimosoid legumes, even in some of the less speciose genera such as the neotropical genus *Stryphnodendron*. This genus includes 35 species occurring from Nicaragua to Southern Brazil mostly in humid forests and savannas. Previous taxonomic studies of *Stryphnodendron* have highlighted morphologically distinct groups within the genus, recognized by differences on leaves (number of pinnae and size of leaflets), inflorescences (a simple or compound thyrse), and fruit types (legume, nucoid legume or follicle). Recent phylogenetic analyses have confirmed the non-monophyly of *Stryphnodendron*, supporting the recognition of three independent and morphologically well-delimited genera. Here we re-circumscribe *Stryphnodendron* and propose the two new genera *Gwilymia* and *Naiadendron*. In addition, we also provide an updated taxonomic account of the closely related genus *Microlobius*, including the proposal of a lectotype for the single species in the genus. *Gwilymia*, Leguminosae, *Microlobius*, *Naiadendron*, *Parapiptadenia*, Phylogeny, Piptadenia group, *Pityrocarpa*, *Pseudopiptadenia*

## ﻿Introduction

Non-monophyly is an issue for several mimosoid legume genera, with relatively few, but significant exceptions as seems to be the case in the genera *Mimosa* L. (Simon et al. 2011) and *Inga* Mill. (Dexter et al. 2017). As well as questioning the characters that were traditionally used to circumscribe mimosoid genera, various molecular phylogenetic studies have revealed the need for new taxonomic circumscriptions of previously large (e.g., *Acacia* Mill.), medium sized (e.g., *Calliandra* Benth.; Souza et al. 2013; *Prosopis* L.; Hughes et al. 2022) and small genera (e.g., *Pseudopiptadenia* Rauschert; Simon et. al. 2016; Borges et al. 2022).

*Stryphnodendron* Mart. currently comprises 35 species mostly distributed in humid forests and savannas of tropical America (Occhioni 1990; Lima et al. 2020; Scalon et al. 2022). The genus has been traditionally distinguished from other genera with diplostemonous flowers (stamens twice the petal number per flower) in tribe Mimoseae (*sensu*Lewis and Elias 1981) by its juvenile spicate inflorescences covered by prophylls and by pinnae with alternate leaflets (Lewis and Elias 1981), as well as by its young shoots covered in reddish granular trichomes and its indehiscent fruits. However, these and other putative diagnostic characters are not exclusive to *Stryphnodendron*, and they vary within the genus (as traditionally circumscribed) as well as across the phylogeny in which the genus is placed (Occhioni-Martins 1981; Guinet and Caccavari 1992; Caccavari 2002; Simon et al. 2016), casting doubts on the genus circumscription.

The recognition of morphologically distinct groups of *Stryphnodendon*, based on the morphology of leaves (number of pinnae and size of leaflets), inflorescences, fruits (Occhioni-Martins 1981; Scalon et al. 2022) and pollen grains (Guinet and Caccavari 1992), has long been known. Phylogenetic studies based on a limited number of plastid and nuclear molecular markers, but including a comprehensive sampling of species, concurred with this view by demonstrating that *Stryphnodendron*, as currently circumscribed, is a polyphyletic assemblage containing three strongly supported lineages (Simon et al. 2016). In addition, the relationships between these three lineages and the closely related genera *Parapiptadenia*, *Pseudopiptadenia* and *Microlobius* remain unresolved (Simon et al. 2016; Ribeiro et al. 2018). The polyphyly of *Stryphnodendron* was recently confirmed by phylogenomic studies, although with a sparser taxonomic sampling (Koenen et al. 2020; Ringelberg et al. 2022), but since these phylogenomic studies did not sample the monospecific *Microlobius*, its phylogenetic position was unclear.

*Microlobius* is here included in the phylogenomic framework depicted by Ringelberg et al. (2022) and this sheds light on its relationship to the different lineages that compose the genus *Stryphnodendron* in its current circumscription. In addition, we combine morphological and phylogenetic evidence to assess the taxonomic limits of *Stryphnodendron*. Based on our results, we propose a narrower circumscription for the genus *Stryphnodendron* by segregating two new genera. In addition, we provide an identification key to the seven genera now recognized within the Stryphnodendron clade, present an updated description of *Microlobius*, and designate a lectotype for the single species in that genus.

## ﻿Materials and methods

### ﻿Phylogenomic analyses

To test the placement of *Microlobius* in a phylogenomic context, we merged transcriptome data for three mimosoid species (*Albiziajulibrissin* Durazz., *Entadaabyssinica* Steud. ex A.Rich., and *Microlobiusfoetidus* (Jacq.) M. Sousa & G. Andrade) generated by Koenen et al. (2020) with a hybrid capture dataset now increased to 997 genes for 63 Caesalpinioid taxa, 33 from Koenen et al. (2020) and 30 from Ringelberg et al. (2022). The hybrid capture dataset contains ten taxa from the Stryphnodendron clade (sensu Koenen et al. (2020)), including three *Stryphnodendron* species, and abundant outgroup sampling across Caesalpinioideae, including 25 taxa from the Albizia clade and nine taxa from the Entada clade (Suppl. material 2: Table S1). As this method combines molecular data from different data sets (transcriptome and hybrid capture), the placement of the *Albiziajulibrissin* and *Entadaabyssinica* transcriptome samples in the final phylogeny serves as confidence tests for the placement of *Microlobiusfoetidus*: if the transcriptome samples of *A.julibrissin* and *E.abyssinica* are placed in the expected place in their correct clades, this suggests that *M.foetidus*, for which only transcriptome data are available, is also placed correctly.

We cleaned raw transcriptome reads using Trimmomatic v. 0.36 (Bolger et al. 2014) with the same settings as used by Nicholls et al. (2015): ILLUMINACLIP:TruSeq3-PE.fa: 2:30:10 LEADING:3 TRAILING:3 SLIDINGWINDOW:4:15 MINLEN:36. Gene assembly was performed with HybPiper (Johnson et al. 2016), using default settings and the updated 997 nuclear MimoBaits sequences (Koenen et al. 2020, Ringelberg et al. 2022) as a target set. Assembled gene sequences of the three transcriptome samples were expressed as DNA sequences by HybPiper. We recovered 991, 956, and 988 genes with at least 75% of the target length for *A.julibrissin*, *E.abyssinica*, and *M.foetidus*, respectively. HybPiper recovers multiple sequences of at least 75% of the target length for a taxon-gene combination; these are flagged as ‘potential paralogs’. Relatively few such potential paralogs (from now on referred to simply as paralogs) were found: 55, 46, and 45, respectively. All sequences, including paralogs, were used in the downstream analyses. At this point the transcriptome sequences (three taxa) and hybrid capture sequences (63 taxa, assembled by Ringelberg et al. 2022) were merged, i.e., transcriptome- and hybrid capture-derived sequences, both expressed as DNA, were pooled across all 66 taxa for each gene. This resulted in a combined dataset with sequences of 997 genes, including all paralogs of both transcriptome and hybrid capture data, which was used in downstream analyses.

We removed outlier sequences, i.e. strongly-divergent sequences placed on very long branches in preliminary gene trees due to orthology assessment or alignment errors, with two rounds of a modified version of the Yang and Smith (2014) pipeline: we aligned all the sequences for each gene with MACSE v. 2.01 (Ranwez et al. 2011), removed sites with a column occupancy < 0.3 with pxclsq (Brown et al. 2017), inferred gene trees using RAxML v. 8.2.12 (Stamatakis 2014) (with the GTRGAMMA model and 200 rapid bootstraps), and removed taxa on long branches with the trim_tips.py script of Yang and Smith (2014), with a relative cut-off of 0.1 and an absolute cut-off of 0.3. In the first round of this approach 181 sequences were removed, out of a total of 66,455 sequences across all genes, and in the second 26, indicating that most outliers, resulting from factors such as alignment errors, have been removed from the 997 gene trees.

We analysed the root-to-tip variance of each of the 997 gene trees with the dist.nodes function of the R (R Core Team 2022) package *ape* (Paradis and Schliep 2019). Four trees with a root-to-tip variance > 0.009 were removed, leaving 993 gene trees. These gene trees were used to generate a species tree with the multi-species coalescent approach using ASTRAL-Pro v. 1.1.6 (Zhang et al. 2020). ASTRAL-Pro was selected because it can use multi-labelled gene trees, i.e. gene trees in which individual taxa may be represented by multiple gene copies, thereby avoiding preliminary orthology assessment. Finally, we used PhyParts (Smith et al. 2015) to assess gene tree support and conflict for each node in the species tree, using the nodes with a bootstrap support of > 50% in the 993 gene trees.

### ﻿Phylogenetic analysis and ancestral state inference

We complemented the phylogenomic analyses described above with the phylogenetic analysis of nuclear (ITS) and plastid (*matK/trnK*, *trnD-T*, *trnL-F*) fragments (White et al. 1990; Taberlet et al. 1991; Möller and Cronk 1997; Hu et al. 2000; Wojciechowski et al. 2004; Simon et al. 2009) for the broader taxon sampling of Simon et al. 2016. The dataset included 96 terminals, of which 49 belonged to *Stryphnodendron* (23 species), two to *Microlobius* (one species), four to *Parapiptadenia* (four species), seven to *Pseudopiptadenia* (five species) and three to *Pityrocarpa* (Benth.) Britton & Rose (three species). Remaining terminals are external groups, and belong to *Anadenanthera* Speg., *Inga*, *Parkia* R. Br., *Piptadenia* Benth., *Mimosa*, *Senegalia* Raf., and *Vachellia* Wight & Arn.

Trees were inferred using a backbone constraint based on the results of the phylogenomic analyses, which included the following relationships: (*Lachesiodendronviridiflorum*, (((*Piptadeniaadiantoides*, *Piptadeniagonoacantha*), (*Mimosamyriadenia*, (*Mimosaceratonia*, *Mimosapigra*))), (((*Stryphnodendronpaniculatum*, *Microlobiusfoetidus*), (*Stryphnodendronpulcherrimum*, *Stryphnodendronadstringens*)), ((*Pseudopiptadeniacontorta*, *Pseudopiptadeniapsilostachya*), (*Stryphnodendronduckeanum*, (*Pityrocarpamoniliformis*, (*Parapiptadeniaexcelsa*, *Parapiptadeniazehntneri*))))))).

Phylogenetic analyses were performed with both maximum parsimony and Bayesian methods. Search parameters for the parsimony analysis, all performed in PAUP* version 4 (Swofford 2003), included two rounds of heuristic search with 1000 replicates of random taxon addition and tree bisection-reconnection branch swap, saving 15 trees per replicate. We estimated branch support using 10000 iterations of bootstrap resampling using the same parameters mentioned above. We used the CIPRES Science Gateway (Miller et al. 2010) implementation of MrBayes version 3.2 (Ronquist et al. 2012) for Bayesian inference. We performed two runs of four chains using a GTR+I+G model for all partitions for 10^7^ generations, sampling trees every 1000 generations. Sampled trees and branch posterior probabilities were summarized on a 50% majority rule tree after discarding the first 25% trees as burn-in.

To infer putative morphological synapomorphies, we optimized 17 morphological characters previously sampled for the group (Simon et al. 2016; http://morphobank.org/permalink/?P2220) onto the resulting Bayesian tree with Mesquite v. 3.70 (Maddison and Maddison 2021). All characters were mapped using parsimony and treated as unordered.

### ﻿Taxonomic analysis

The taxonomic updates that we present here are based on taxon observations made during field expeditions and on examination of specimens from the following herbaria (acronyms according to Thiers 2018): ALCB, B, BHCB, BM, BOTU, BR, CEN, CEPEC, CESJ, CPAP, CVRD, E, ESA, F, G, GUA, HB, HEPH, HRB, HRCB, HTO, HUEFS, HUFU, IAC, IAN, IBGE, INPA, IPA, K, M, MBM, MG, MO, NY, OUPR, OXF, P, R, RB, RFA, SP, SPF, SPSF, U, UB, US, UEC, UFG, UFMS, VIC, W, WU.

We follow Scalon et al. (2022) and Harris and Harris (2001) for habit, indumentum, and leaf terminology; Weberling (1989) for inflorescence and flower terminology; and Barroso et al. (1999) for fruits. The geographical distribution maps were made using SimpleMappr (Shorthouse 2010).

## ﻿Results and discussion

### ﻿Placement of *Microlobius* and *Stryphnodendron* polyphyly

**Figure 1.**
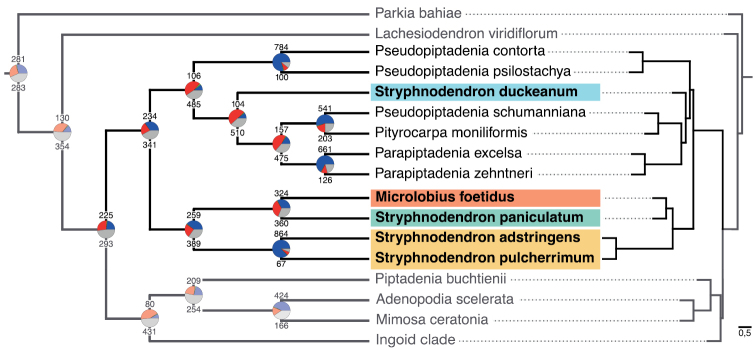
Phylogeny of the Stryphnodendron clade based on combined transcriptome and hybrid capture data. Left: Cladogram with pie charts depicting support and conflict per bipartition across 993 individual gene trees; blue sections indicate support, green sections support for the most common conflicting topology, red sections support for alternative conflicting topologies, and gray sections uninformative gene trees. Numbers above and below pie charts are numbers of supporting and conflicting gene trees, respectively. Right: Tree with internal branch lengths expressed in coalescent units, and terminal branches assigned an arbitrary uniform length.

Our phylogenomic analysis places *Microlobius* in a clade together with all *Stryphnodendron* species, except for *Stryphnodendronduckeanum* (Fig. 1). While this placement is not supported by all gene trees, the most likely alternative topology is far less common among the gene trees (Fig. 1). This suggests that most gene tree conflict found across the phylogeny (Suppl. material 1: Fig. S1) most likely reflects a lack of signal for particular nodes among many of the gene trees, rather than strong support for alternative topologies (Koenen et al. 2020, Ringelberg et al. 2022).

The combination of transcriptome- and hybrid capture-based samples in a single phylogenetic analysis is validated by placing of the two outgroup transcriptome samples in the resulting phylogeny (Suppl. material 1: Fig. S1). *Entadaabyssinica* is placed within *Entada* in the sister clade of *Elephantorrhiza* (Burch.) Skeels, matching the *matK* phylogeny of LPWG (2017). *Albiziajulibrissin* is resolved as the sister to *A.umbellata* (Vahl) E.J.M. Koenen in *Albizia* s.s., in accordance with unpublished data of Koenen et al.

**Figure 2.**
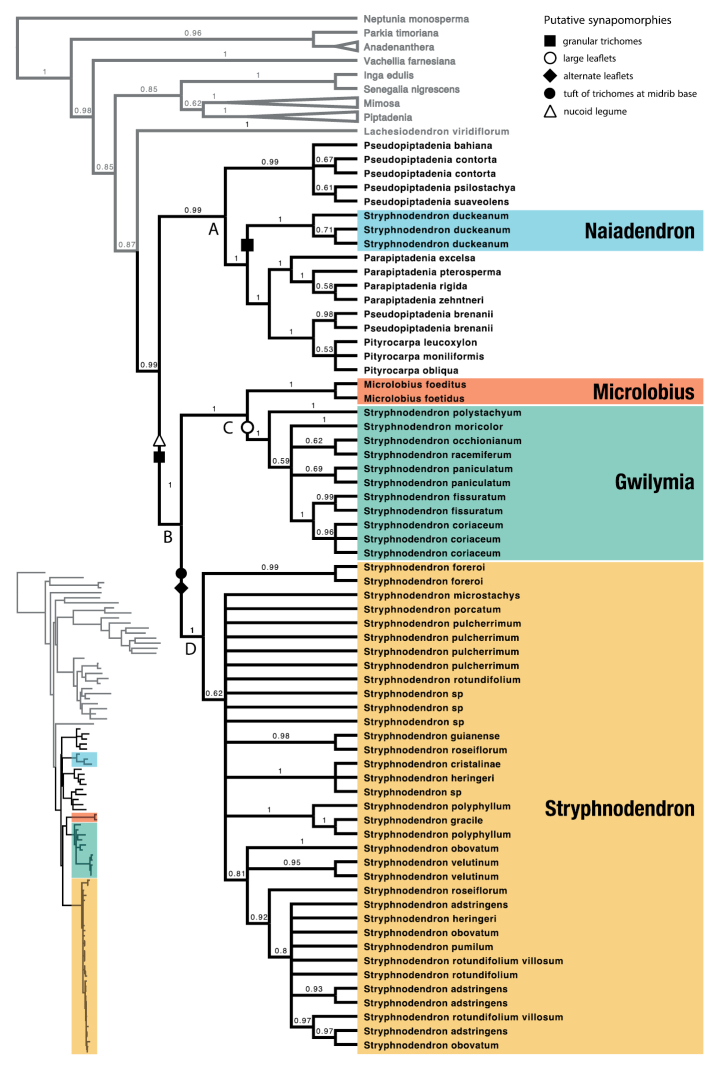
Relationships in the *Stryphnodendron* clade based on (*matK/trnK*, *trnD-trnT*, *trnL-trnF*) and nuclear (ITS) DNA data; constrained by a phylogenomic backbone. 50% majority-rule consensus tree and posterior probability values (above branches) from trees sampled in the posterior Bayesian analysis. Symbols indicate selected putative morphological synapomorphies. The inset tree depicts the Bayesian phylogram with inferred branch lengths.

The constrained parsimony and Bayesian analyses match the phylogenomic data and expands the relationships by presenting a denser taxonomic sampling. *Stryphnodendron* was recovered as a polyphyletic assemblage and its species group in three highly supported lineages: (1) *S.duckeanum* appears isolated from the remainder of the genus in a clade with representatives of the genera *Parapiptadenia, Pityrocarpa* and *Pseudopiptadenia* (clade A); (2) *Microlobiusfoetidus* was supported as sister to a clade including seven species of *Stryphnodendron* (clade C); and (3) a main *Stryphnodendron* lineage (Clade D; Fig. 2).

Some of these relationships are supported by putative morphological synapomorphies (Fig. 2). Indehiscent fruits (nucoid legumes) and granular reddish trichomes support clade C, which includes *Microlobius* and the majority of *Stryphnodendron* sensu lato species (excluding *S.duckeanum*). Although changes from nucoid fruits to follicles occur (including in *Microlobius*), the nucoid legume is inferred as a synapomorphy for this group. Contrary to previous results (Simon et al. 2016), reddish granular trichomes are supported as having independent origins in *S.duckeanum* and the clade including *Microlobius* and the remaining *Stryphnodendron* species. Large leaflets are a synapomorphy for the *Stryphnodendron* lineage which is sister to *Microlobius* in clade C. Alternate leaflets and a tuft of trichomes at the base of the midrib, traits commonly associated with *Stryphnodendron*, support clade D that represents the main lineage of the genus. No studied morphological character was recovered as a synapomorphy of clade C, which includes *Microlobius* and *Gwilymia*. The remaining characters (Suppl. material 1: Figs S2–S18) are either too homoplastic or not informative in the context of *Stryphnodendron* polyphyly.

Given the phylogenetic evidence presented above and the morphological distinctiveness and diagnosability of the three *Stryphnodendron* lineages and *Microlobius*, we propose to split *Stryphnodendron* into three distinct genera: (1) the new genus *Gwilymia*, which includes mostly Amazonian species bearing leaves with few pinnae and large opposite leaflets, inflorescence usually a compound thyrse, and fruit a nucoid legume; (2) the new and monospecific Amazonian genus *Naiadendron* with long petiolar nectaries, opposite leaflets, and non-septate, papery legumes, more similar to the fruits of *Piptadenia* than to any other species of *Stryphnodendron* or *Gwilymia*; and (3) a re-circumscribed *Stryphnodendrons.str.*, which includes species with multipinnate leaves and small alternate leaflets (e.g., *S.adstringens* (Mart.) Coville, the type species of the genus), and the inflorescence a simple thyrse. In addition, we maintain *Microlobius*, which is sister to *Gwilymia*, as a monospecific genus with branches and leaves with a strong garlic odour, petiolar nectary absent, a few pairs of pinnae and opposite leaflets, and fruit a follicle.

**Figure 3.**
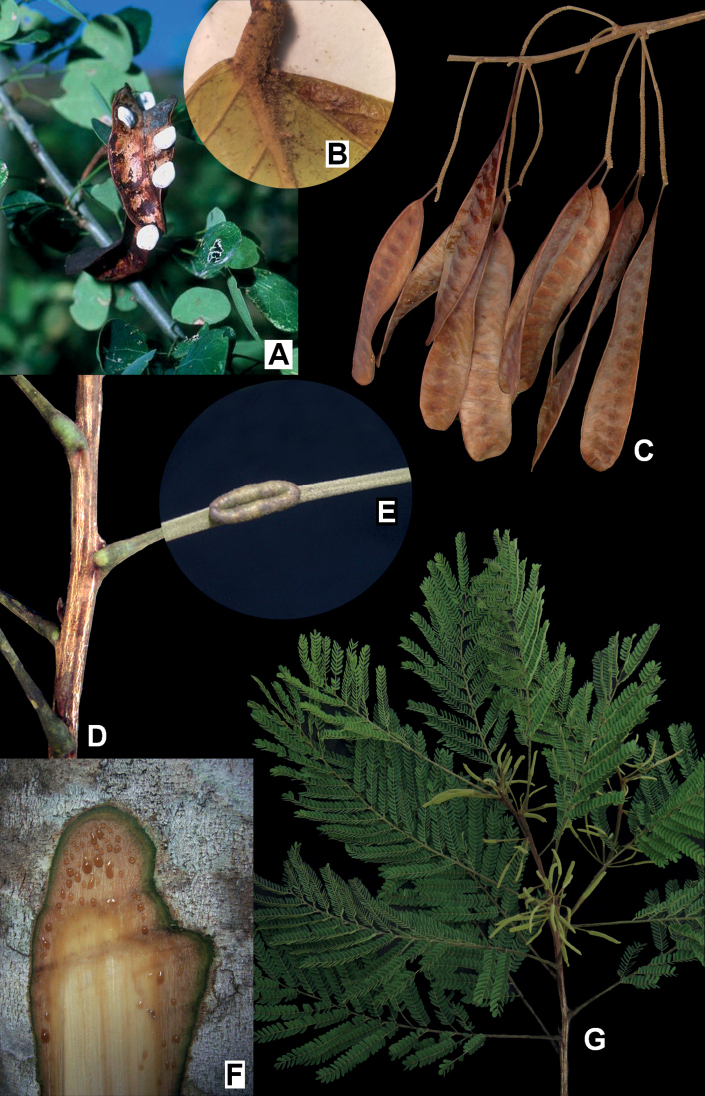
**A, B***Microlobiusfoetidus*: **A** fruiting branch with white seeds exposed **B** detail of a leaflet showing the tuft of trichomes at the base of the midrib **C–G***Naiadendronduckeanum*: **C** fruits **D** detail of the striated branch **E** detail of petiolar nectary (upper view, magnified) **F** bark slash showing reddish exudate **G** flowering branch. Photos: **A** Donovan Bailey **B** Alexandre Gibau de Lima **C–G** Marcelo Simon.

**Figure 4.**
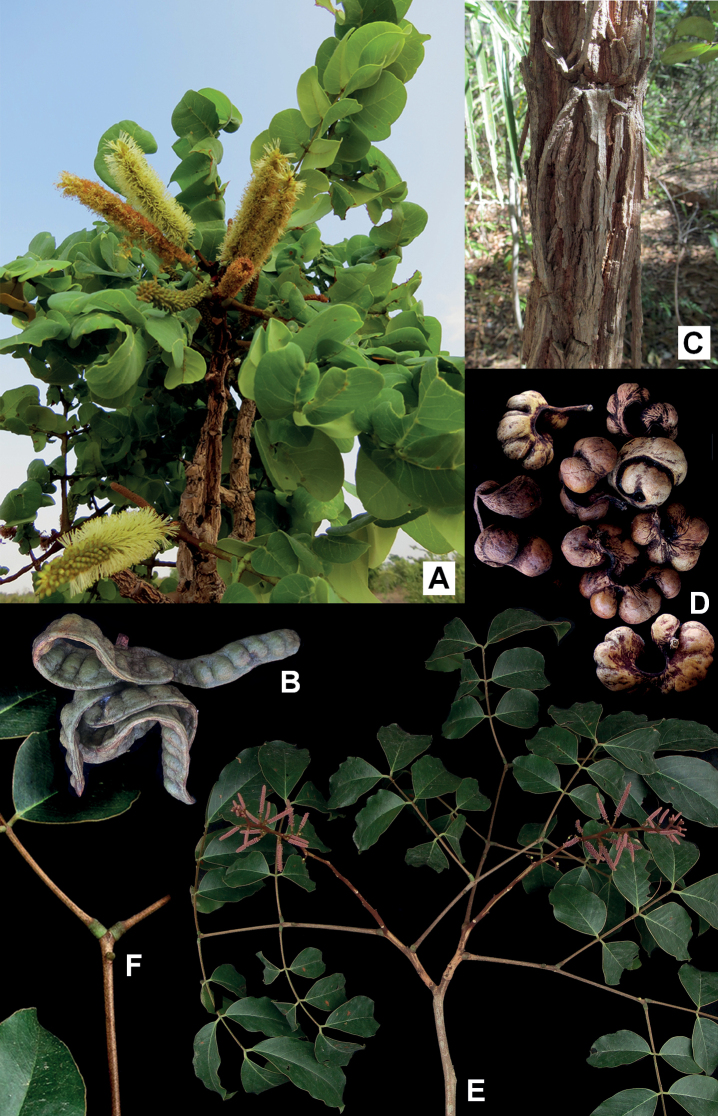
**A, B***Gwilymiacoriacea*: **A** flowering branch **B** fruit **C, D***G.fissurata*: **C** detail of bark **D** fruit **E, F***G.paniculata*: **E** flowering branch with young inflorescences **F** detail of the extrafloral nectary on the leaf rachis. Photos: Marcelo Simon.

**Figure 5.**
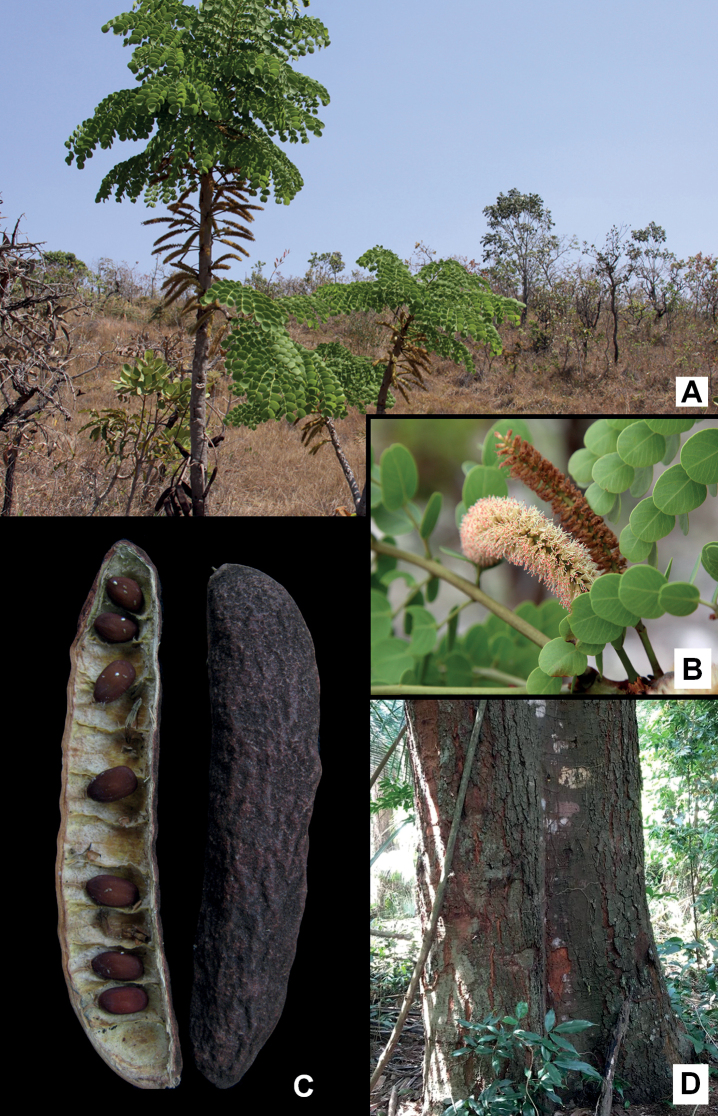
**A, C***Stryphnodendronadstringens*: **A** habit **B** foliage and inflorescences **C** fruit (manually opened) and seeds **D***S.flavotomentosum*: trunk and detail of bark. Photos: **A, B** Henrique Moreira **C** Marcelo Simon **D** Geovane Siqueira.

**Figure 6.**
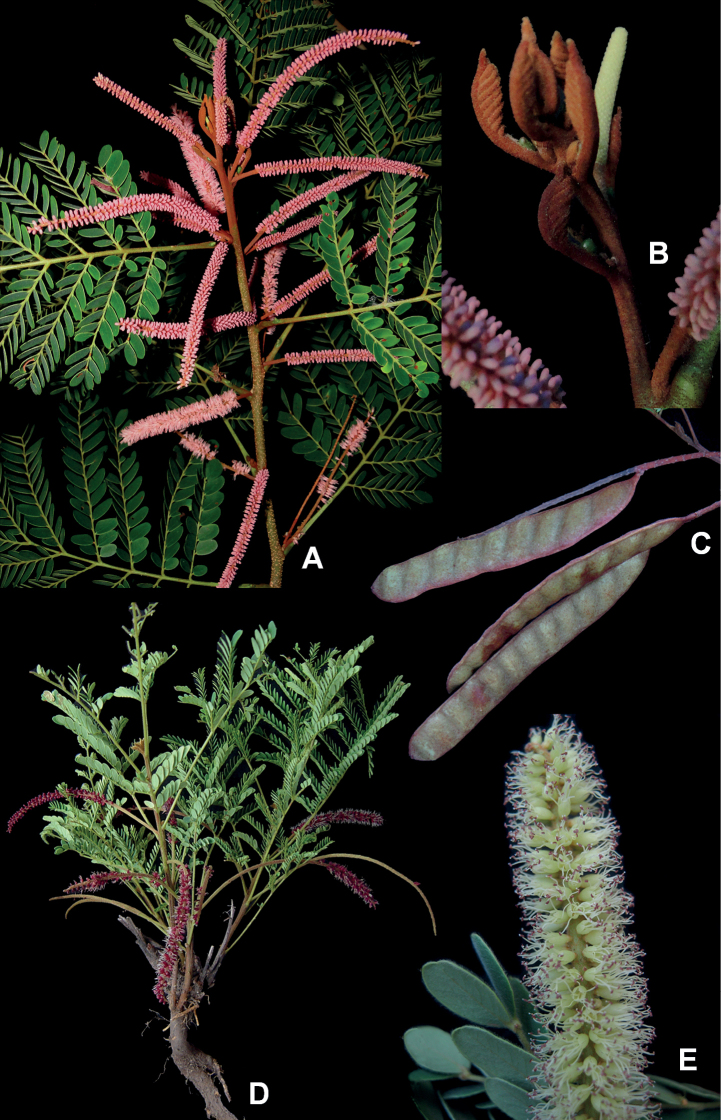
**A, B***Styphnodendronforreroi*: **A** flowering branch **B** detail of young shoot covered by reddish granular trichomes **C, D***S.heringeri*: **C** fruits **D** habit **E***S.rotundifolium*: detail of inflorescence. Photos: Marcelo Simon.

An alternative to the circumscription proposed above would be not to describe a new genus and instead to merge *Microlobius* into *Stryphnodendron* (excluding *S.duckeanum*). Although this option would result in fewer taxonomic changes (a single species of *Microlobius* being transferred to *Stryphnodendron* vs. seven new combinations in *Gwilymia*), the marked morphological distinctiveness and easy diagnosability of the *Stryphnodendron* and *Gwilymia* lineages support their recognition as different genera (Figs 3–6; Table 1).

**Table 1.** Diagnostic characters of the four Stryphnodendroid lineages. * *Microlobius* was not sampled in Guinet and Caccavari 1992; a description provided in a later work includes its single species (Caccavari 2002) which suggests that the genus might have its own distinct pollen type.

Character	*Microlobius*	*Gwilymia*	*Naiadendron*	*Stryphnodendron*
Garlic odour evident in branches and leaves	Present	Absent	Absent	Absent
Length of petiolar nectary (mm)	Nectary absent	0.5–2	8–12	0.5–2
Number of pairs of pinnae	1–2 (–3)	2–4 (–6)	10–22	(3–) 5–32
Insertion of leaflets	Opposite	Opposite	Opposite	Alternate
Size of leaflets (cm)	2–5 × 1–2.5	2.5–16 × 1.5–8	0.6–1.2 × 0.3–0.5	0.6–1.2 × 0.3–0.6
Tuft of trichomes on leaflets	Present or absent	Absent	Absent	Usually present
Type of Inflorescence	Simple thyrse	Compound thyrse (diplothyrsi or pleiothyrsi), except *G.coriacea* and *G.fissurata*	Simple thyrse	Simple thyrse
Fruit type	Follicle	Nucoid legume (indehiscent)	Legume (dehiscent along both margins)	Nucoid legume (indehiscent) or follicle
Fruit texture	Coriaceous	Coriaceous or woody	Chartaceous	Coriaceous or woody
Seed colour	White	Brown or ochre	Ochre	Brown or ochre
Pollen type (Guinet and Caccavari 1992)	*	*S.fissuratum*, *S.coriaceum* and *S.polystachyum* types	*S.adstringens* type	*S.adstringens*, *S.microstachyum* and *S.piptadenioides* types

In addition, the circumscription adopted here preserves the morphological distinctiveness of *Microlobius* regarding both *Stryphnodendron* and *Gwilymia* (presence or absence of a garlic odour and petiolar nectary, number of pairs of pinnae, insertion of leaflets, type of inflorescence, type of fruit, and the color of the seeds) as well as the ecological identity of the groups since *Microlobius* is the only member of clade B inhabiting seasonally dry vegetation, whereas *Gwilymia* and *Stryphnodendron* are restricted to humid forests and savannas (Figs 3–6; Table 1).

## ﻿Taxonomy

### ﻿Key to the genera of the Stryphnodendron clade (sensu Koenen et al. 2020, Ringelberg et al. 2022, Borges et al. 2022)

**Table:** 

1	Young branches and leaves lacking ferruginous granular trichomes	**2**
–	Young branches and leaves covered with ferruginous granular trichomes	**4**
2	Fruit a legume, dehiscing along both margins; flowers with reddish petals and stamens	***Parapiptadenia***
–	Fruit a follicle, dehiscing along one margin only; flowers with greenish petals and whitish stamens	**3**
3	Extrafloral nectary between or just below the first pair of pinnae; spikes isolated in the axil of the coeval leaf; fruits moniliform, with deeply constricted margins, and with thick coriaceous and pubescent valves	***Pityrocarpa***
–	Extrafloral nectary between the base and the middle of the petiole; spikes clustered in terminal efoliate pseudoracemes or below the coeval leaves; fruits with a linear or oblong body, straight or shallowly sinuous margins and thin to thick woody and glabrous valves	***Marlimorimia***
4	Branches and leaves with a strong garlic odour; leaves with 1–2 (–3) pairs of pinnae, each pinna comprising a single pair of leaflets, extrafloral nectary absent on the petiole and on the branches; inflorescence a spike, 3–6 cm long (peduncle and rachis); fruit 4–7 × 1–1.5 cm; seeds white	***Microlobius***
–	Branches and leaves without a garlic smell; leaves always with more than one pair of pinnae, each pinnae comprising 3 or more pairs of leaflets, extrafloral nectary present on the petiole or, in *Gwilymiacoriacea* and *G.fissurata*, on the branch directly below the insertion of the petiole; inflorescence a spike, 3.5–20 cm long (peduncle and rachis); fruit 8–14 × 2–3.5 cm; seeds brown or ochre	**5**
5	Leaves with 2–4(–6) pairs of pinnae; leaflets 2.5–16 × 1.5–8 cm; inflorescence a compound thyrse (except in *Gwilymiacoriacea* and *G.fissurata* which have a simple thyrse)	***Gwilymia***
–	Leaves with (3–)5–32 pairs of pinnae; leaflets 0.6–1.2 × 0.3–0.6 cm; inflorescence always a simple thyrsi	**6**
6	Branches not striate; petiolar nectary 0.5–2 mm long; leaflets alternate, abaxial surface with a tuft of trichomes at the base of the midrib; petals cohered for at least ½ of their length; fruit coriaceous or woody and indehiscent (a nucoid legume) or splitting along a single margin (a follicle)	***Stryphnodendron***
–	Branches strongly striate; petiolar nectary ca. 10 mm long; leaflets opposite, without a tuft of trichomes on the abaxial surface; petals cohered for only ⅓ of their length; fruit chartaceous, dehiscent along both margins (a legume)	***Naiadendron***

#### ﻿1. Microlobius

Taxon classificationPlantaeFabalesFabaceae

C. Presl, Abh. Königl. Böhm. Ges. Wiss. ser. 5, 3: 496. 1845.

BAD05C0A-4F37-5EF3-93EB-21B12510ABD8

Goldmania Rose, Mém. Soc. Phys. Genève 34: 274. 1903. Type. Goldmaniaplatycarpa Rose [= Microlobiusfoetidus (Jacq.) M. Sousa & G. Andrade].

##### Type.

*Microlobiusmimosoides* C. Presl [= *Microlobiusfoetidus* (Jacq.) M. Sousa & G. Andrade]

##### Description.

**Trees** or shrubs, 3–10 m tall; branches unarmed, smooth, lenticellate, glabrescent, sparsely covered with ferruginous granular trichomes, with a strong garlic odour (hence the epithet of its single species). **Stipules** caducous. **Leaves** bipinnate, petiole glabrescent, sparsely covered with ferruginous granular trichomes, petiolar nectary absent; rachis (0.2–) 3–7 cm long, glabrous or sparsely pubescent, sparsely covered with ferruginous granular trichomes, nectaries 1–3, 0.5–0.8 mm long, patelliform, inserted between the pairs of pinnae; pinnae in 1–2 (–3) opposite pairs, pinnae rachillae nectaries 1–2, 0.3 mm long, patelliform, positioned close to the pair of leaflets; leaflets in 1–2 opposite pairs, 2–5 × 1–2.5 cm, obovate or sometimes elliptic, a tuft of trichomes sometimes present at the base on the abaxial surface. **Inflorescence** a simple thyrse formed by cymules of 2–5 spikes, these 3–6 cm long (including the peduncle and rachis), covered with ferruginous granular trichomes, spike prophyll caducous, flower prophyll usually persistent during anthesis. **Flowers** monoclinous; calyx pentamerous, gamosepalous, 0.8–1 mm long, campanulate, pubescent; corolla pentamerous, gamopetalous, 3–4 mm long, cohered for at least ½ of its length, narrow-campanulate, pubescent; androecium with 10 stamens, anthers with a caducous apical gland. **Fruit** a follicle, sessile or subsessile, 4–7 × 1–1.5 cm, subfalcate, sparsely covered with ferruginous granular trichomes, valves coriaceous, dark brown. **Seeds** obovate, white. Fig. 3.

##### Geographic distribution and habitat.

**Figure 7.**
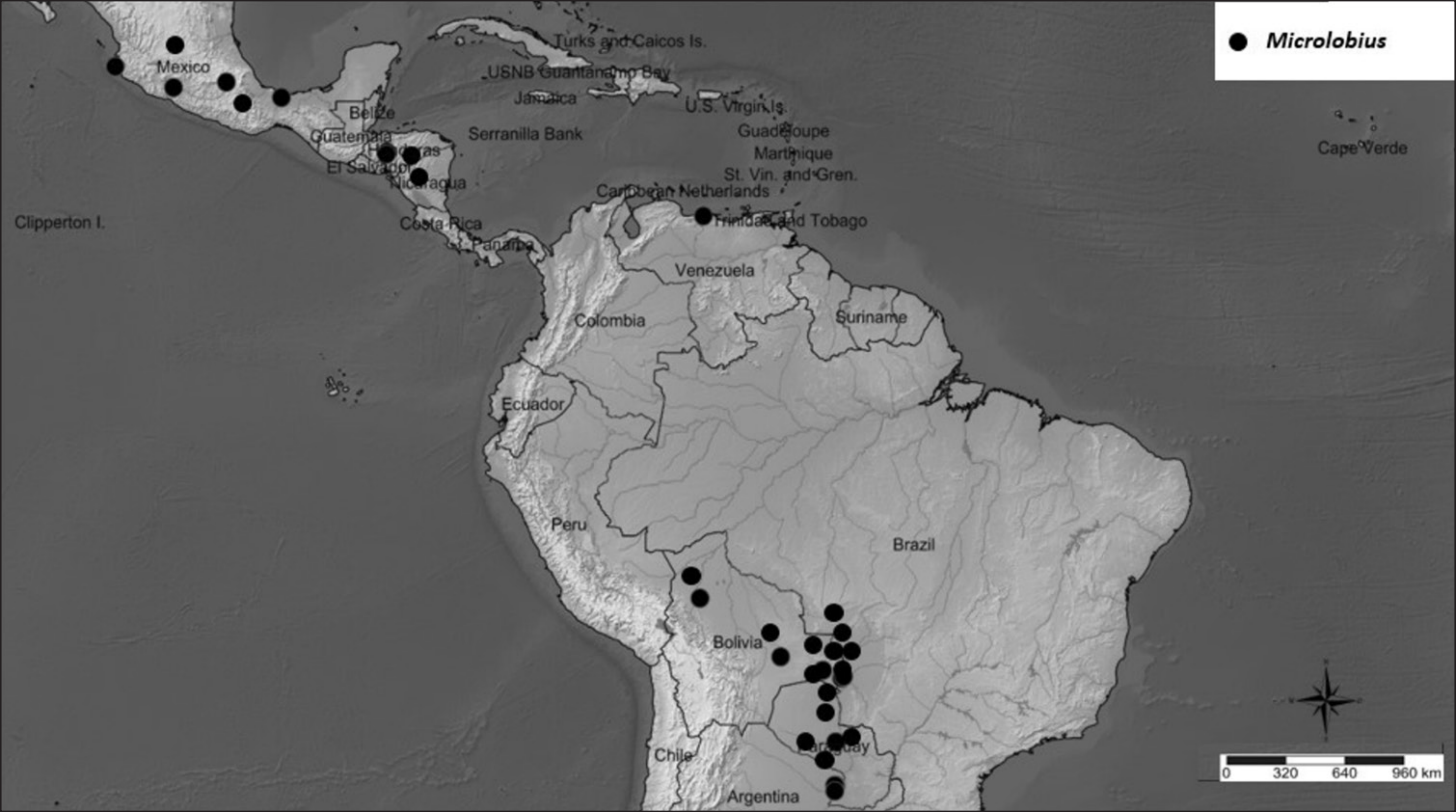
Distribution of *Microlobiusfoetidus*.

A monospecific genus distributed in seasonally dry forests of Mexico, Honduras, Venezuela, Brazil, Bolivia, Paraguay and Argentina (Fig. 7).

##### Etymology.

From *micro*- (small) and *lobion*- (pods) in reference to the relatively small fruits, a noteworthy characteristic of *Microlobius* compared to closely related genera.

#### ﻿1.1 Microlobius foetidus

Taxon classificationPlantaeFabalesFabaceae

(Jacq.) M. Sousa & G. Andrade, Anales Inst. Biol. Univ. Nac. Autón. México, Bot. 63(1): 104. 1992.

301E8C39-1CDC-53C0-A2F6-F7CC36117492

Mimosa foetida Jacq., Pl. Hort. Schoenbr. 3: 73. 1798. Type. [illustration] “Mimosafœtida T. 390” in Jacquin, Pl. Hort. Schoenbr. 3, t. 390. 1798 (lectotype, designated here).
Inga foetida (Jacq.) Willd., Sp. Pl. Editio quarta 4(2): 1008. 1806.
Acacia foetida (Jacq.) Kunth, Nov. Gen. Sp. (quarto ed.) 6: 265. 1823.
Piptadenia foetida (Jacq.) Benth., Trans. Linn. Soc. London 30(3): 366. 1875.
Goldmania foetida (Jacq.) Standl., Contr. U.S. Natl. Herb. 23(2): 354. 1922.
Microlobius mimosoides C. Presl, Abh. Königl. Böhm. Ges. Wiss. ser. 5, 3: 497. 1845. Type. Mexico. *Habitat in Mexico*, 1791, *Haenke s.n.* (holotype: PRC 452782!).
Goldmania platycarpa Rose, Mém. Soc. Phys. Genève 4: 274. 1903. Type. Mexico, Culiacan, Sinaloa, 19 Mar 1899, *E.A. Goldman 371* (holotype: US360292! [catalog] US00001026! [barcode], isotype: GH00066208!).
Piptadenia platycarpa (Rose) J.F. Macbr., Contr. Gray Herb. 59: 18. 1919.

##### Notes.

The protologue of *Mimosafoetida* (“*crescit in India Occidentali. In caldario floret Junio & Julio*”) suggests that Jacquin had the plant growing in a heated greenhouse in the gardens of Schönbrunn Palace. However, it is not possible to know whether he based his description on a dried specimen from the Americas or on the plant cultivated in Vienna. According to Stafleu and Cowan (1979), Jacquin “certainly made herbarium material of Austrian plants and plants in the gardens under his care” and they “are present in small numbers in a number of herbaria”. His West Indies samples (which were acquired by Sir Joseph Banks), however, are very difficult to locate and it is not known if Jacquin made sizeable collections there; his specimens in the Banks herbarium (BM) are rare and consist of fragmentary specimens (Stafleu and Cowan 1979). The origin of the seeds that arrived in Vienna is also questionable, as there are currently no records of the species occurring in the Antilles, and the seeds were most probably gathered in eastern Mexico. We were unable to find any specimen that could be recognized as a type in the herbaria listed by Stafleu and Cowan (1979) and other collections, confirming Sousa and Andrade´s (1992) previous searches (“*holotipo W, no encontrado*”). For this reason, we select the colored plate accompanying the description of the species as the lectotype of *Mimosafoetida*.

Based on variable features and a very small sample of South American plants, Sousa and Andrade (1992) recognized the North/Central and South American disjunct populations of the genus as two subspecies (Fig. 7). It is not our objective to evaluate infraspecific taxa, so we opted to maintain the circumscription of *Microlobiusfoetidus* as currently accepted.

**1.1.1 Microlobiusfoetidus(Jacq.)M. Sousa & G. Andradesubsp.foetidus .**

#### ﻿1.1.2 Microlobius foetidus subsp. paraguensis

Taxon classificationPlantaeFabalesFabaceae

(Benth.) M. Sousa & G. Andrade, Anales Inst. Biol. Univ. Nac. Autón. México, Bot. 63(1): 106. 1992.

97B9425A-5704-5F0C-83E2-6C9D4B958FE7

Goldmania paraguensis (Benth.) Brenan, Kew Bull. 10(2): 178. 1955.
Piptadenia quadrifolia N.E. Br., 20: 53. 1894. Trans. & Proc. Bot. Soc. Edinburgh. Type. Paraguay. Rio Pilcomayo expedition, a small tree abundant in the isolated patches of monte around Fortin Page, 01 Sep 1890, *J.G. Kerr 1* (holotype: K000504735!).

##### Basionym.

*Pithecellobiumparaguense* Benth., Trans. Linn. Soc. London 30(3): 574. 1875.

##### Type.

Paraguay. Monte Claro, 10 Jun 1858, *M. Gibert 39* (holotype: K000504734!). *Piptadeniaparaguensis* (Benth.) Lindm., Bih. Kongl. Svenska Vetensk.-Akad. Handl. 24(3/7): 36. 1898.

##### Notes.

Stafleu and Cowan (1976) mentioned that Gibert´s collections are distributed in several European, Argentine and Uruguayan herbaria, but we only found a single specimen of *M. Gibert 39*, housed at K. Since the Kew Herbarium includes that of Bentham, we indicate this specimen as the holotype of *Pithecellobiumparaguense*. Many South American herbaria, which are still not digitized, may house Gibert’s collections, including isotypes of *P.paraguense*.

#### ﻿2. Gwilymia

Taxon classificationPlantaeFabalesFabaceae

A.G. Lima, Paula-Souza & Scalon gen. nov.

9EB09117-0130-5284-A54A-F179E6E92749

urn:lsid:ipni.org:names:77303770-1

##### Type.

*Gwilymiapaniculata* (Poepp. & Endl.) A.G. Lima, Paula-Souza & Scalon ≡ *Stryphnodendronpaniculatum* Poepp. & Endl., Nov. Gen. Sp. Pl. 3: 81. 1845).

##### Diagnosis.

*Gwilymia* is similar to *Microlobius*, but it differs in having branches and leaves without a garlic odour (*vs.* a strong garlic odour in *Microlobius*); leaves with 2–4 (–6) pairs of pinnae (*vs.* 1–2 pairs of pinnae); each pinna with at least 3 pairs of leaflets (*vs.* a single pair of leaflets); extrafloral nectary present on the petiole or, in *G.coriacea* and *G.fissurata*, on the branch directly below the insertion of the petiole (*vs.* extrafloral nectary absent on the petiole and on the branch); inflorescence usually a compound thyrse (*vs.* always a simple thyrse); spikes 4–20 cm long (*vs.* 3–6 cm long); fruit an indehiscent (nucoid) legume 12–14 × 2–2.5 cm (*vs.* a follicle 6–7 × 1–1.5 cm), and brown or ochre seeds (*vs.* white seeds). *Gwilymia* also resembles *Stryphnodendron*, but it differs in leaves with 2–4 (–6) pairs of pinnae (*vs.* (3–) 5–32 pairs of pinnae in *Stryphnodendron*), opposite leaflets, 2.5–16 × 1.5–8 cm (*vs.* alternate, 0.6–1.2 × 0.3–0.6 cm), inflorescence usually a compound thyrse (*vs.* always a simple thyrse).

##### Description.

**Trees** 2.5–40 m tall. **Branches** unarmed, not odoriferous, smooth, usually lenticellate, young shoots and leaves glabrescent, pubescent, or tomentose and covered with reddish granular trichomes. **Stipules** caducous. **Leaves** bipinnate, petiolar nectary 1 (absent in *G.coriacea* and *G.fissurata*), 0.5–2 mm long, conical, lenticular or verruciform, positioned at the base or apex of the petiole; rachis 7–23 cm long, rachis nectaries 1–4, 0.5–2.5 mm long, conical, lenticular, patelliform or verruciform, inserted between the pairs of pinnae or just below them; pinnae in 2–4 (–6) opposite or subopposite pairs, rachillae nectaries 1–5, patelliform or verruciform, inserted between or just below the distal pairs of leaflets; leaflets in 3–5 opposite pairs, 2.5–16 × 1.5–8 cm, broadly-oblong, elliptic, ovate or obovate, not odoriferous, no tuft of trichomes at the midrib base. **Inflorescence** a compound thyrse (diplothyrsi or pleiothyrsi, a simple thyrse in *G.coriacea* and *G.fissurata*), cymules in 2–5 spikes, spike 4–20 cm long (including peduncle and rachis), covered with ferruginous granular trichomes, inflorescence prophyll persistent (caducous in *G.coriacea* and *G.fissurata*), floral bracts usually persistent. **Flowers** monoclinous; calyx pentamerous, gamosepalous, ca. 0.5–1 mm long, campanulate, cupuliform or tubular, puberulent or pubescent; corolla pentamerous, gamopetalous, 2–5 mm long, cohered for at least ½ of its length, campanulate or tubular, glabrous, pubescent, or tomentose; stamens 10, anthers with a caducous apical gland. **Fruit** an indehiscent, nucoid legume, sessile, 12–14 × 2–2.5 cm, curved, falcate or spiralled (straight to slightly curved in *G.moricolor* and *G.racemifera*), laterally-compressed or sub-turgid, sparsely covered with ferruginous granular trichomes, valves woody or coriaceous, brown. **Seeds** elliptic, obovate, or orbicular, brown or ochre. Fig. 4.

##### Geographic distribution and habitat.

**Figure 8.**
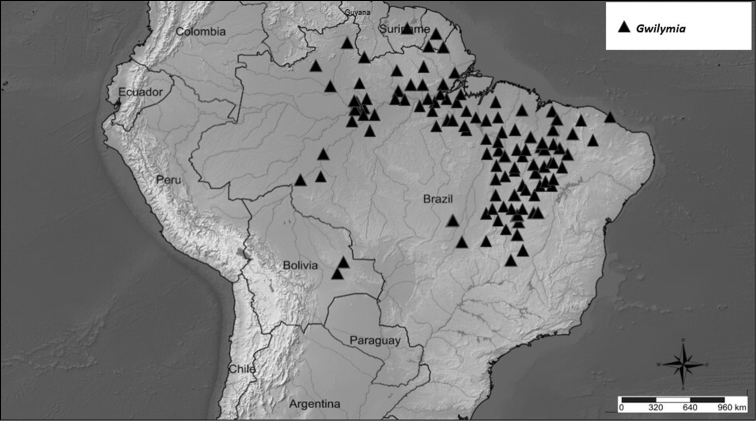
Distribution of *Gwilymia*.

*Gwilymia* species occur in the Amazon rainforest, seasonal forests and savannas of Bolivia, Brazil, French Guiana, Guyana, Suriname and Venezuela (Fig. 8).

##### Etymology.

*Gwilymia* honors Dr. Gwilym Peter Lewis, one of the Royal Botanic Gardens Kew’s most prominent botanists for his exceptional contributions to the advance of legume systematics.

##### Notes.

*Gwilymia* comprises seven species formerly placed in *Stryphnodendron*, all of which have 2–4 (–6) pairs of pinnae, opposite leaflets, 2.5–16 × 1.5–8 cm, compound thyrses (except in *G.coriacea* and *G.fissurata*), and nucoid (indehiscent) legumes.

#### ﻿2.1 Gwilymia coriacea

Taxon classificationPlantaeFabalesFabaceae

(Benth.) A.G. Lima, Paula-Souza & Scalon comb. nov.

B5BE96B6-6C8B-5139-82EE-3F196258A98D

urn:lsid:ipni.org:names:77303771-1 

##### Basionym.

*Stryphnodendroncoriaceum* Benth., Trans. Linn. Soc. London 30(3): 373. 1875.

##### Type.

Brazil. Minas Gerais. “Fermoso provinciae Minas Geraes”, s.d., *Martius 1820* (lectotype: M 0218783!, designated by Scalon et al. 2022; isolectotypes: F!, M!, MO!, NY!).

#### ﻿2.2 Gwilymia fissurata

Taxon classificationPlantaeFabalesFabaceae

(E.M.O. Martins) A.G. Lima, Paula-Souza & Scalon comb. nov.

94E3F716-A98E-55E4-9DDD-F4E1C39BD4DF

urn:lsid:ipni.org:names:77303772-1

##### Basionym.

*Stryphnodendronfissuratum* E.M.O. Martins, Revista Brasil. Biol. 40(4): 730. 1980.

##### Type.

Brazil. Mato Grosso, “Habitat ad Município Barra do Garças, 265 km NNE de Xavantina, Serra do Roncador”, s.d., *G. Eiten & L. Eiten 8956* (holotype: SP 129687!, isotypes: NY!, K!).

#### ﻿2.3 Gwilymia moricolor

Taxon classificationPlantaeFabalesFabaceae

(Barneby & J.W. Grimes) A.G. Lima, Paula-Souza & Scalon comb. nov.

D34CDBA5-0B6E-52AB-8F21-8E2E65151A49

urn:lsid:ipni.org:names:77303773-1

##### Basionym.

*Stryphnodendronmoricolor* Barneby & J.W. Grimes, Brittonia 36(1): 45. 1984.

##### Type.

French Guiana. Saül, Monts La Fumée, 22 Nov 1982, *Mori & Boom 15236* (holotype: P 00077203! [transferred from CAY], isotypes: NY!, P 00710285!).

#### ﻿2.4 Gwilymia occhioniana

Taxon classificationPlantaeFabalesFabaceae

(E.M.O. Martins) A.G. Lima, Paula-Souza & Scalon comb. nov.

092EDB78-D5AC-5539-BDCC-CE4F03FF0010

urn:lsid:ipni.org:names:77303774-1

##### Basionym.

*Stryphnodendronocchionianum* E.M.O. Martins, Leandra 2(2): 121. 1972.

##### Type.

Brazil. Pará, Rodovia Belém–Brasília km 306, 10 Mar 1960, *Oliveira 997* (holotype: IAN 106945!, isotypes: NY!, UB!).

#### ﻿2.5 Gwilymia paniculata

Taxon classificationPlantaeFabalesFabaceae

(Poepp. & Endl.) A.G. Lima, Paula-Souza & Scalon comb. nov.

F1A92395-4F28-5467-8366-D7715C29F6D9

urn:lsid:ipni.org:names:77303775-1

Piptadenia poeppigii Klotzsch ex Benth., Trans. Linn. Soc. London 30(3): 367. 1875.
Stryphnodendron rizzinianum E.M.O. Martins, Leandra 6(7): 92. 1975. Type. Brazil. Amazonas, Borba, “Habitat in silva ad flumen Madeira”, 07 Nov 1935, *Ducke s.n.* (holotype: RB 29044!, isotypes: K!, OXF!, NY!, U!, *pro parte*, US!).

##### Basionym.

*Stryphnodendronpaniculatum* Poepp. & Endl., Nov. Gen. Sp. Pl. 3: 81. 1845.

##### Type.

Brazil. “Crescit in sylvis primaevis flumini Amazonum conterminis circum Ega [Tefé]”, Nov 1834, *Poeppig 2783* (lectotype: W 0048790!, designated by Scalon et al. 2022; isolectotypes: G!, NY!, OXF!, P!, W 0048789!).

#### ﻿2.6 Gwilymia polystachya

Taxon classificationPlantaeFabalesFabaceae

(Miq.) A.G. Lima, Paula-Souza & Scalon comb. nov.

B1E1E558-9262-5FF0-80E9-D5EA0862B4D5

urn:lsid:ipni.org:names:77303776-1

Stryphnodendron polystachyum (Miq.) Kleinhoonte, Recueil Trav. Bot. Néerl. 22: 416. 1926.
Piptadenia tocantina Ducke, Arch. Jard. Bot. Rio de Janeiro 4: 33. 1925. Type. Brazil. Pará, “Habitat in silva primaria non inundata infra stationen Arumateua viae ferreae Alcobacensis in regione fluminis Tocantins civitate Pará”, 14 Jul 1916, *Ducke s.n.* (holotype: MG 16252!, isotypes: G!, K!, P!, RB!).

##### Basionym.

*Piptadeniapolystachya* Miq., Linnaea 18: 590. 1845.

##### Type.

Suriname, “Crescit prope Bergendaal”, September, *collector unknown s.n*. (holotype: U 52627–A!).

#### ﻿2.7 Gwilymia racemifera

Taxon classificationPlantaeFabalesFabaceae

(Ducke) A.G. Lima, Paula-Souza & Scalon comb. nov.

508DD329-D8FA-5CE5-B7CA-001FED3FCDEA

urn:lsid:ipni.org:names:77303779-1

Stryphnodendron racemiferum (Ducke) W.A. Rodrigues, Ciência e Cultura 21(2): 438. 1969.

##### Basionym.

*Piptadeniaracemifera* Ducke, Arch. Jard. Bot. Rio de Janeiro 5: 124. 1930.

##### Type.

Brazil. Amazonas, Maués, Rio Curuçá, 16 Dec 1927, *Ducke s.n.* (holotype: RB 20188!; isotypes: U!, US!).

#### ﻿3. Naiadendron

Taxon classificationPlantaeFabalesFabaceae

A.G. Lima, Paula-Souza & Scalon gen. nov.

E45463E1-2760-50ED-BA19-A6A69D51A81B

urn:lsid:ipni.org:names:77303777-1

##### Type.

*Naiadendronduckeanum* (Occhioni f.) A.G. Lima, Paula-Souza & Scalon ≡ *Stryphnodendronduckeanum* Occhioni f., Revista Brasil. Biol. 19: 209. 1959).

##### Diagnosis.

*Naiadendron* is closely related to *Stryphnodendron*, but it differs in having strongly striate branches (*vs.* smooth or only slightly striate in *Stryphnodendron*), a petiolar nectary 8–12 mm long (*vs.* 0.5–2 mm long), leaflets inserted in opposite pairs (*vs.* alternate pairs), fruit a legume, valves dehiscing along both sutures (*vs.* fruit an indehiscent, nucoid legume or follicle). The genus differs from *Piptadenia* in having unarmed branches (*vs.* armed branches in *Piptadenia*) and ferruginous granular trichomes on branches and leaves (*vs.* ferruginous granular trichomes absent).

##### Description.

**Trees** 8–30 m tall; branches unarmed, strongly striate, castaneous, apex yellow-tomentose and covered with ferruginous granular trichomes, not odoriferous. **Stipules** caducous. **Leaves** bipinnate, petiole yellow-puberulent or yellow-tomentulose, sparsely covered with ferruginous granular trichomes, petiolar nectary 1, 8–12 mm long, narrowly oblong, positioned at the base of the petiole; rachis 10–23 cm long, yellow-puberulent or yellow-tomentulose, sparsely covered with ferruginous granular trichomes, rachis nectary 1, ca. 2 mm long, oblong, inserted below the distal pair of pinnae; pinnae in 10–22 subopposite to opposite pairs, rachilla nectary 1, 1 × 0.4 mm, oblong, secretory, inserted below the distal pair of leaflets; leaflets in 15–23 opposite pairs, 0.6–1.2 × 0.3–0.5 cm, oblong, elliptic or sometimes obovate, no tuft of trichomes at the base on the abaxial surface, not odoriferous. **Inflorescence** a simple thyrse formed by cymules of 3–5 spikes, spike 4–7 cm long (peduncle plus rachis), covered with ferruginous granular trichomes, spike prophyll caducous, flower prophyll usually caducous. **Flowers** monoclinous; calyx pentamerous, gamosepalous, ca. 0.5 mm long, campanulate, puberulent; corolla pentamerous, gamopetalous, 1.8–2 mm long, cohered for ⅓ of its length, narrow-campanulate, yellow-tomentulose; androecium with 10 stamens, anthers with a caducous apical gland. **Fruit** a legume (dehiscent along both margins), peduncle 1.3–2 cm long, fruit body 12–15 × 2–2.5 cm, linear to narrow-oblong, laterally-compressed sparsely covered with ferruginous granular trichomes, chartaceous, brown. **Seeds** obovate to elliptic, ochre colored. Fig. 3.

##### Geographic distribution and habitat.

**Figure 9.**
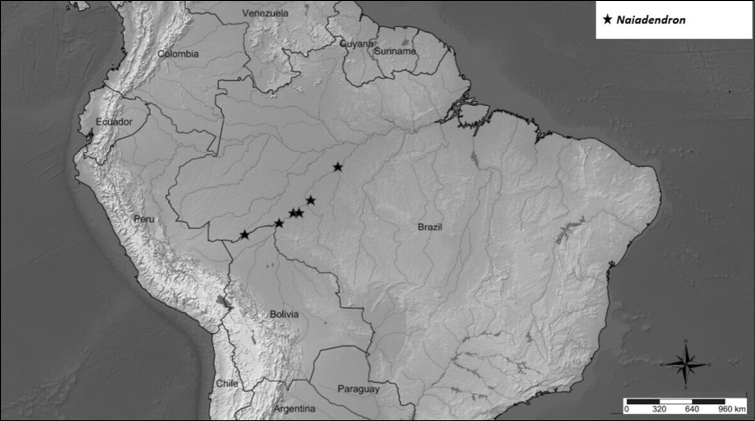
Distribution of *Naiadendronduckeanum*.

*Naiadendron* is endemic to the Amazon rainforest, being recorded from the Brazilian states of Acre, Amazonas and Rondônia. It grows on clay or sandy soil in ombrophilous and *terra firme* forests (Fig. 9).

##### Etymology.

The name *Naiadendron* celebrates the Amazon rainforest and the legacy of Carl Friedrich Philipp von Martius (1794–1868), who named the Brazilian Amazon after the Naiads, Greek mythology’s nymphs of freshwater.

##### Notes.

Strongly striate branches, a petiolar nectary 8–12 mm long, and the fruit a legume (valves dehiscing along both margins) are the main diagnostic morphological characteristics of *Naiadendron*.

Occhioni (1959) described *Stryphnodendronduckeanum*, based only on flowering specimens, and pointed out its morphological similarity to *S.guianense*. However, both morphological (Scalon 2007; Lima et al. 2021; Scalon et al. 2022) and phylogenetic evidence (Simon et al. 2016; Ribeiro et al. 2018) have indicated that *S.duckeanum* should be recognized as an independent taxon, now named as the new genus *Naiadendron*.

#### ﻿3.1 Naiadendron duckeanum

Taxon classificationPlantaeFabalesFabaceae

(Occhioni) A.G. Lima, Paula-Souza & Scalon comb. nov.

7AE75D5A-D665-566C-8739-BD1A8677CDF0

urn:lsid:ipni.org:names:77303778-1

##### Basionym.

*Stryphnodendronduckeanum* Occhioni, Revista Brasil. Biol. 19: 209. 1959.

##### Type.

Brazil. Rondônia, Porto Velho, Rio Madeira, Amazonas, 09 Jun 1936, *Ducke s.n.* (lectotype: RFA 11684!, designated by Scalon et al. 2022; isolectotype: US!).

#### ﻿4. Stryphnodendron

Taxon classificationPlantaeFabalesFabaceae

Mart., Flora 20(2): Beibl. 117. 1837.

90C98A00-F5C4-5E29-A196-D5FACC86654C

Folianthera Raf., Sylva Tellur.: 120. 1838. Type. Foliantheraguianensis (Aubl.) Raf. [= Stryphnodendronguianense (Aubl.) Benth.].

##### Type.

*Stryphnodendronbarbadetiman* (Vell.) Mart. [= *Stryphnodendronadstringens* (Mart.) Coville].

##### Description.

**Trees, shrubs, or subshrubs**, 0.25–45 m tall; branches unarmed, smooth or slightly striate, usually lenticellate, glabrescent, pubescent, tomentose, velutinous or villous, apex covered with ferruginous granular trichomes, not odoriferous. **Stipules** usually caducous **Leaves** bipinnate, petiole glabrescent, pubescent, tomentose, velutinous or villous, covered with ferruginous granular trichomes, petiolar nectary 1, 0.5–2 mm long, verruciform, conical, fusiform, lenticular or patelliform, positioned at the base or sometimes at the apex of the petiole; rachis 10–25 cm long, glabrescent, pubescent, tomentose, velutinous or villous, ferruginous-pulverulent, rachis nectaries 1–5, 0.5–3 mm long, conical, lenticular, patelliform or verruciform, inserted between the pairs of pinnae or just below them; pinnae in (3–) 5–32 subopposite, opposite or rarely alternate pairs, rachilla nectaries 1–5, conical, patelliform or verruciform, inserted between or just below the distal pairs of leaflets, leaflets in 8–20 alternate pairs, 0.6–1.2 × 0.3–0.6 cm, oblong, elliptic or sometimes obovate, a tuft of trichomes usually present at the base on the abaxial surface, not odoriferous. **Inflorescence** a simple thyrse formed by cymules of 2–6 spikes, spike 7–18 cm long (including peduncle and rachis), covered with ferruginous granular trichomes, spike prophyll caducous, flower prophyll usually caducous. **Flowers** monoclinous or rarely diclinous (only staminate flowers observed), calyx pentamerous, gamosepalous, 0.5–1 mm long, campanulate, cupuliform or tubular, glabrous, pubescent, puberulent, ciliate, tomentose, or villous; corolla pentamerous, gamopetalous 2.5–5 mm long, cohered for at least ½ of its length, campanulate, cupuliform or tubular, glabrous, pubescent, puberulent, tomentulose, tomentose, or villous; androecium with 10 stamens, anthers with apical gland caducous. **Fruit** a nucoid legume (indehiscent) or follicle, sessile, 8–14 × 2–3.5 cm, linear, oblong, or slightly curved, laterally compressed or turgid, sparsely covered with ferruginous granular trichomes, valves woody or coriaceous, brown. **Seeds** obovate to elliptic, black, brown, or ochre. Figs 5, 6.

##### Geographic distribution and habitat.

**Figure 10.**
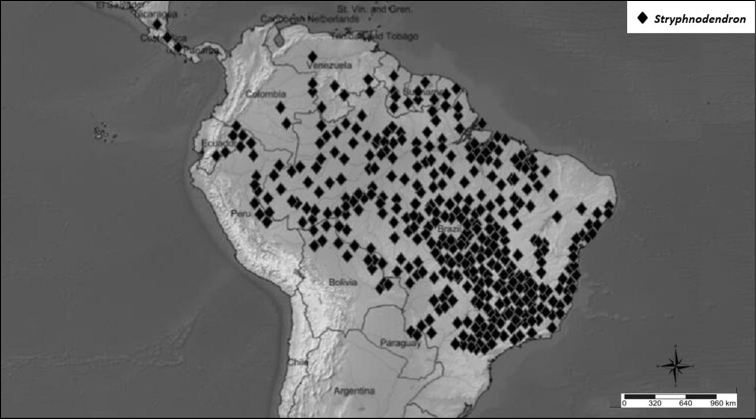
Distribution of *Stryphnodendron*.

*Stryphnodendron* is a neotropical genus with its northern limit in Nicaragua and southern limit in the Brazilian state of Paraná. *Stryphnodendron* species occur in several vegetation types, and are especially frequent in savannas and in the Amazonian forest (Fig. 10).

##### Etymology.

The name *Stryphnodendron* comes from *stryphnos*- (adstringent) and *dendron*- (tree) and is a reference to the astringent properties of its tannin-rich bark.

##### Notes.

*Stryphnodendron* was first described by Martius (1837) based on three species: *S.barbadetiman* (Vell.) Mart., *S.polyphyllum* Mart. and *S.rotundifolium* Mart. The genus subsequently received a more detailed description and a broader circumscription by Bentham (1841, 1875, 1876), and currently comprises 28 species.

The genus can be recognized by a suite of characters: unarmed branches, ferruginous granular trichomes on young shoots and leaves, caducous stipules, leaves with (3–)5–32 pairs of pinnae; leaflets 0.6–1.2 × 0.3–0.6 cm, inflorescence always a simple thyrse, and the fruit a nucoid (indehiscent) legume or follicle.

*Stryphnodendron* differs from *Microlobius* in having branches and leaves lacking a garlic odour (vs. branches and leaves with a strong garlic odour in *Microlobius*), leaves with (3–)5–32 pairs of pinnae (vs. leaves with 1–2 (–3) pairs of pinnae), alternate leaflets (vs. opposite leaflets), an extrafloral nectary present on the petiole (vs. extrafloral nectary absent on the petiole), brown or ochre seeds (vs. white seeds). The morphological distinctiveness and diagnosability among *Stryphnodendron*, *Gwilymia* and *Naiadendron* are addressed above.

#### ﻿4.1 Stryphnodendron adstringens

Taxon classificationPlantaeFabalesFabaceae

(Mart.) Coville, Century Dict. 11: 111. 1910.

612601CB-23C2-5345-90F3-4E6182744D36

Mimosa barbadetiman Vell., Fl. Flumin. Icon. 11: 7. 29 Oct 1831. Type. [icon ined.] “Polyg. Monoec.: MIMOSA barbadetimao Tab. 7” (Manuscript Sect. of Torre do Tombo, Lisbon PT-TT-MSLIV-2780_m0021; icon ined. copy in Manuscript Sect., Bibliot. Nac., Rio de Janeiro No. I-17, 06, 001, mss1198660_011. Lectotype, designated by Scalon et al. 2022).
Stryphnodendron barbadetiman (Vell.) Mart., Flora 20(2): Beibl. 117. 1837 (“*barbatiman*”).

##### Basionym.

*Acaciaadstringens* Mart., Reise Bras. 2: 548. 1828.

##### Type.

Brazil. Minas Gerais. “Habitat in campus agrestibus, Minas Geraes, Serro Frio ad Tejuco et alibi parfim”, May, *Martius s.n.* (holotype: M 0218791!).

#### ﻿4.2 Stryphnodendron barbatulum

Taxon classificationPlantaeFabalesFabaceae

Rizzini & Heringer, Revista Brasil. Biol. 47(3): 449. 1987.

96E81300-DB33-584C-B996-4F69B8291736

Stryphnodendron sallesianum Heringer & Rizzini, Revista Brasil. Biol. 47: 450. 1987. Type. Brazil. Distrito Federal, Brasília, Barragem do Torto, 11 Nov 1985, *Salles 388* (holotype: RB 288834!, isotype: RB!).

##### Type.

Brazil. Distrito Federal, Brasília, Barragem do Torto, 14 Sep 1985, *Salles & Heringer 241* (holotype: RB 288833!).

#### ﻿4.3 Stryphnodendron confertum

Taxon classificationPlantaeFabalesFabaceae

Heringer & Rizzini, Anais Acad. Brasil. Ci. 38(Suppl.): 104. 1966.

236B7424-C93E-58FA-B4F5-1B22D87F583B

##### Type.

Brazil. Distrito Federal. Brasília, Parque Nacional de Brasília, 10 Sep 1963, *Heringer 9178* (holotype: RB 118803!, isotypes: HB!, K!, M!, NY!, RFA!, UB!).

#### ﻿4.4 Stryphnodendron conicum

Taxon classificationPlantaeFabalesFabaceae

Scalon, Phytotaxa 544(3): 237. 2022.

CE742864-0F24-5974-B16C-5D4978D7F3EA

##### Type.

Brazil. Pará, Oriximiná, Área de Mineração Rio Norte, 5 km da vila residencial, 1°28'S, 56°23'W, 11 Nov 1987, *C.A. Cid Ferreira 9548* (holotype: INPA 155605!, isotypes: F!, K!, MO!, NY!, RB!, US!).

#### ﻿4.5. Stryphnodendron cristalinae

Taxon classificationPlantaeFabalesFabaceae

Heringer, Anais Acad. Brasil. Ci. 40: 234. 1968.

CF1B88C0-6E81-56C4-A4DD-0DD5A5CE4BE0

Stryphnodendron campestre Forero, Brittonia 24(2): 143. 1972. Type. Brazil. Goiás, “Serra dos Christaës”, 1818, *Pohl 847* (holotype: NY00003371!, isotypes: F!, MO!, W!).

##### Type.

Brazil. Goiás, Cristalina, elev. 1350 m, 15 Aug 1967, *E.P. Heringer 11182* (holotype: RB 132217!, isotypes: HB! K! MG! UB!).

#### ﻿4.6 Stryphnodendron dryaticum

Taxon classificationPlantaeFabalesFabaceae

Scalon, Phytotaxa 544(3): 240. 2022.

4BB23B8A-5A8C-5755-A01D-ACFAA2B4363D

##### Type.

Brazil. Rio de Janeiro, Macaé, estrada para Glicério, ca. 2 km do Córrego do Ouro, 42°04'W, 22°13'S, 23 Jun 1987, *Lima et al. 2988* (holotype: RB 265629!, isotype: MBM!).

#### ﻿4.7 Stryphnodendron excelsum

Taxon classificationPlantaeFabalesFabaceae

Harms, Repert. Spec. Nov. Regni Veg. 19(4–7): 64. 1923.

FCE2F099-31D7-5B1D-9267-411D65B414B9

##### Type.

Costa Rica. Atlant. Küste, Savannen und Wälder am Rio Hondo, elev. 150–300 m, Jun 1903, *Pittier 16997* (lectotype: G 00367833!, designated by Scalon et al. 2022; isolectotypes: US!, NY!).

#### ﻿4.8 Stryphnodendron flavotomentosum

Taxon classificationPlantaeFabalesFabaceae

A.G. Lima & V.C. Souza, Syst. Bot. 46(1): 70. 2021.

9AF045CB-8619-5990-BA5C-C064D9A40278

##### Type.

Brazil. Espírito Santo, Baixo Guandú, Fazenda Galiléia, no barranco do rio próximo a estrada do Mutum Preto em Baixo Guandu, lado esquerdo, 11 Dec 1991, *D.A. Folli 1519* (holotype: ESA 108191!, isotypes: CVRD!, VIES!).

#### ﻿4.9 Stryphnodendron foreroi

Taxon classificationPlantaeFabalesFabaceae

E.M.O. Martins, Contr. Univ. Michigan Herb. 14: 83. 1980.

86062263-AC4A-57B6-B0B3-3301CD9C42BF

##### Type.

Brazil. Rondônia, track from Mutumparaná to rio Madeira, 30 Nov 1968, *Prance et al. 8995* (holotype: MG 039652!, isotypes: F!, NY!, R!, S!, US!).

#### ﻿4.10 Stryphnodendron glandulosum

Taxon classificationPlantaeFabalesFabaceae

(Forero) Scalon, Phytotaxa 544(3): 245. 2022.

A1451D52-6667-5E42-B5B0-85CCA434F885

##### Basionym.

Stryphnodendronguianense(Aubl.)Benth.subsp.glandulosum Forero, Brittonia 24(2): 145. 1972.

##### Type.

Brazil. Pará, “Museu Paraense, Cult. et Peruvia orientalis (Rio Huallaga J. Huber anno 1898)”, Sep 1936, *A. Ducke 274* (holotype: NY 00003368!, isotypes: K!, R!, US!).

#### ﻿4.11 Stryphnodendron gracile

Taxon classificationPlantaeFabalesFabaceae

Heringer & Rizzini, Anais Acad. Brasil. Ci. 38(Suppl.): 105. 1966.

29451D6D-D983-599D-AEF8-E098233CA41E

##### Type.

Brazil. Minas Gerais, Serra do Cipó, 12 Nov 1959, *Heringer 7361* (lectotype: RB00584092!, designated by Scalon et al. 2022; isolectotypes: NY!, UB!).

#### ﻿4.12 Stryphnodendron guianense

Taxon classificationPlantaeFabalesFabaceae

(Aubl.) Benth., Trans. Linn. Soc. London 30(3): 374. 1875.

F5B0156B-6DF9-5D31-885B-D2BFF948D055

Acacia guianensis (Aubl.) Willd., Sp. Pl. 4(2): 1061. 1806.
Folianthera guianensis (Aubl.) Raf., Sylva Tellur. 120. 1838.
Piptadenia guianensis (Aubl.) Benth., J. Bot. (Hooker) 4(30): 335. 1841.
Stryphnodendron purpureum Ducke, Arch. Jard. Bot. Rio de Janeiro 1(1): 16. 1915. Type. Brazil. “Alcobaça ad fluvium Tocantins, in sylvis secundariis terrae argillosae rubrae valde frequens”, 28 Dec 1914, *Ducke s.n.* (holotype: MG 15556!, isotypes: BM!, G!, S!, US!).

##### Basionym.

*Mimosaguianensis* Aubl., Hist. Pl. Guiane 2: 938. 1775.

##### Type.

French Guiana, “Habitat in sylvis Caïenna & Guiana”, s.d., *Aublet s.n.* (holotype: BM001135589!).

#### ﻿4.13 Stryphnodendron heringeri

Taxon classificationPlantaeFabalesFabaceae

Occhioni f., Bol. Mus. Bot. Kuhlmann 8(1): 63. 1985.

C60863A3-B960-5DA6-B2FC-FA1D5BE04610

##### Type.

Brazil. Goiás, Alto Paraíso de Goiás, a ca. 87 km ao N da cidade, 30 Oct 1979, Equipe IBGE [“*Heringer*”] *2636* (holotype: IBGE 15208!, isotypes: HB!, K!, MO!, NY!, RB!, UEC!).

#### ﻿4.14 Stryphnodendron holosericeum

Taxon classificationPlantaeFabalesFabaceae

Scalon, Phytotaxa 544(3): 247. 2022.

05CC86D6-EB66-54BD-8A4D-313280D72579

##### Type.

Brazil. Minas Gerais, Formoso, Parque Nacional Grande Sertão Veredas, margem esquerda do Rio Preto, 05 Nov 1989, *Walter et al. 510* (holotype: RB 375879!, isotypes: ESA! IBGE!, K!, RFA!).

#### ﻿4.15 Stryphnodendron levelii

Taxon classificationPlantaeFabalesFabaceae

R.S. Cowan, Mem. New York Bot. Gard. 10(1): 144. 1958.

ABC84C10-5841-598F-8A77-01C4645686C6

##### Type.

Venezuela. Ter. Fed. Amazonas, Cano Guazuriapana, Rio Atabapo near San Fernando de Atabapo, 16 May 1954, *Level 104* (holotype: NY 3369!, isotype: F!, K!, US!, VEN).

#### ﻿4.16 Stryphnodendron microstachyum

Taxon classificationPlantaeFabalesFabaceae

Poepp. & Endl., Nov. Gen. Sp. Pl. 3: 82. 1845.

4DF6C11B-3205-5EBF-9083-07582AAB2FCD

##### Type.

Brazil. “Crescit in sylvis primaevis flumini Amazonum conterminis circum Ega [Tefé]”, Oct 1831, *Poeppig 2738* (holotype: W 0002775!).

#### ﻿4.17 Stryphnodendron orinocense

Taxon classificationPlantaeFabalesFabaceae

Scalon, Phytotaxa 544(3): 252. 2022.

614B30EB-0163-594F-B6D6-E71D22E17F02

##### Type.

Venezuela. Território Amazonas, Rio Orinoco, along left bank of river just below mouth of Rio Ventuari, 125–150 m, 16 Jun 1959, *Wurdack & Adderley 42999* (holotype: IAN 114608!, isotypes: F!, K!, NY!, U!, US!).

#### ﻿4.18 Stryphnodendron platycarpum

Taxon classificationPlantaeFabalesFabaceae

Scalon,Phytotaxa 544(3): 254. 2022.

9C5BD2C7-E0B9-569C-B31F-92250CE83AC3

##### Type.

PERU. Loreto, Requena, bosque inundable, ca. 800 m de la Base Yarina, margen derecha del caño Yarina, en la Zona Reservada del río Pacaya, margen izquierda del Río Ucayali, 22 Mar 1977, *Encarnación E–1071* (holotype: G 0252076!, isotypes: K!, US!).

#### ﻿4.19 Stryphnodendron platyspicum

Taxon classificationPlantaeFabalesFabaceae

Rizzini & Heringer, Anais Acad. Brasil. Ci. 38(Suppl.): 106. 1966.

97CD2915-C65A-5470-9156-530AFAFA60D6

Stryphnodendron pumilum Glaz., Bull. Soc. Bot. France 53 Mem. 3b: 177. 1906, *opus utiq. oppr.*

##### Type.

Brazil. Distrito Federal, Brasília, “Crescit ad campos in Goiás”, 5 Nov 1961, *Heringer 8733* (holotype: RB 113247!, isotypes: HB!, R!, UB!).

#### ﻿4.20 Stryphnodendron polyphyllum

Taxon classificationPlantaeFabalesFabaceae

Mart., Flora 20(2): Beibl. 117. 1837.

5C560745-07D0-5D08-98B5-B1C720058322

##### Type.

Brazil. Minas Gerais, “Minas”, s.d., *Martius 1102* (lectotype: M 0218780!, designated by Scalon et al. 2022; isolectotypes: BR!, G!, K!, P!).

#### ﻿4.21 Stryphnodendron porcatum

Taxon classificationPlantaeFabalesFabaceae

D.A. Neill. & Occhioni f., Ann. Missouri Bot. Gard. 76(1): 357. 1989.

542E865C-ACE5-5C0D-BF49-80FEE6F43DD0

##### Type.

Ecuador. Napo, 1 km N of Coca, 00°25'S, 77°00'W, 15 Sep 1986, *Neill & Palacios 7359* (holotype: QCNE 233!, isotypes: G!, INPA!, K!, MO!, NY!, RFA!, US!).

#### ﻿4.22 Stryphnodendron procerum

Taxon classificationPlantaeFabalesFabaceae

Scalon, Phytotaxa 544(3): 260. 2022.

48997B7F-E042-5198-A88B-8A3390AA5591

##### Type.

Brazil. Amazonas, Maraã, Rio Japurá, margem esquerda, Lago Maraã, 29 Oct 1982, *Amaral et al. 232* (holotype: INPA 106613!, isotypes: K!, MG!, MO!, NY!, UB!, US!).

#### ﻿4.23 Stryphnodendron pulcherrimum

Taxon classificationPlantaeFabalesFabaceae

(Willd.) Hochr., Bull. New York Bot. Gard. 6(21): 274. 1910.

C1927447-0317-561C-801B-9A37CA6564E2

Mimosa pulcherrima (Willd.) Poir., Encycl., Suppl. 1(1): 66. 1810.
Piptadenia foliolosa Benth., J. Bot. (Hooker) 4(30): 336. 1841. Type. Brazil. Amazonas river, s.d., *Poeppig 2776* (lectotype: F0360538F!, designated by Scalon et al. 2022).
Stryphnodendron floribundum Benth., J. Bot. (Hooker) 4(31): 343. 1841. Type. Brazil. s.d., *Gardner 986* (lectotype: K 000090447!, designated by Scalon et al. 2022; isolectotypes: BM!, E!, G!, GH!, NY!, OXF!, P!).
Stryphnodendron angustum Benth., Trans. Linn. Soc. London 30(3): 375. 1875. Type. Brazil. Amazonas, “prope Barra do Rio Negro”, s.d., *Martius Obs. 2758 / Obs. 2578* (lectotype: M 0218774!, designated by Scalon et al. 2022; isolectotypes: M 0218773!, M 0218775!, M 0218776!).
Stryphnodendron melinonis Sagot, Ann. Sci. Nat., Bot., sér. 6, 13: 322. 1882. Type. Guiana Francesa, “in sylvis Maroni”, s.d., *Mélinon s.n.* (lectotype: P 00199449!, designated by Scalon et al. 2022; isolectotypes: BM!, E!, F!, K!, P 00199447! P 00199448!).
Stryphnodendron guianense f. floribundum (Benth.) Ducke, Arch. Jard. Bot. Rio de Janeiro 4: 250. 1925.
Piptadenia cobi Rizzini & A. Mattos, Anais Acad. Brasil. Ci. 40: 233. 1966. Type. Brazil. Bahia, Oct 1939, Menezes [“*Moisés*”] *135* (holotype: RB 55432!, isotype: K!).

##### Basionym.

*Acaciapulcherrima* Willd., Sp. Pl. 4(2): 1061. 1806.

##### Type.

Brazil. “Habitat in provincia Para Brasiliae”, s.d., *Hoffmannsegg s.n.* (holotype: B-W 19136!).

#### ﻿4.24 Stryphnodendron riparium

Taxon classificationPlantaeFabalesFabaceae

Scalon, Phytotaxa 544(3): 265. 2022.

F3B9D799-170D-5014-9B4B-824AA2F033FB

Stryphnodendron inaequale Benth., Trans. Linn. Soc. London 30(3): 374. 1875, *pro syn.*

##### Type.

Brazil. Amazonas, Rio Solimões, ca. 1 km ao sul da Vila Careiro, 23 Aug 1973, *C.C. Berg et al. 1971*1 (holotype: INPA 43195!, isotypes: F!, K!, MG!, MO!, NY!, R!, RFA!).

#### ﻿4.25 Stryphnodendron roseiflorum

Taxon classificationPlantaeFabalesFabaceae

(Ducke) Ducke, Bol. Tecn. Inst. Agron. N. 2: 8. 1944.

3C0A15FC-9735-5968-9F48-8A63F177D79F

##### Basionym.

Stryphnodendronguianense(Aubl.)Benth.subsp.guianensevar.roseiflorum Ducke, Arch. Jard. Bot. Rio de Janeiro 6: 15. 1933.

##### Type.

Brazil. Amazonas, “Frequens in sylvis secundariis siccioribus circa Manaos”, 22 Jun 1929, *Ducke s.n.* (lectotype: RB 10406/ 00540075!, designated by Occhioni-Martins 1981; isolectotypes: G!, K!, US!).

#### ﻿4.26 Stryphnodendron rotundifolium

Taxon classificationPlantaeFabalesFabaceae

Mart., Flora 20(2): Beibl. 117. 1837.

0E36C796-18F5-5D3C-A569-C1DE5464B3F4

##### Type.

Brazil. Piauí, “Oeiras, Prov. Piauhy”, s.d., *Martius s.n.* (holotype: M 0218772!).

#### ﻿4.26.1 Stryphnodendron rotundifolium Mart. var. rotundifolium

Taxon classificationPlantaeFabalesFabaceae

.

AFBFD144-52B8-53B6-B4DF-7F98B4581FDA

Stryphnodendron discolor Benth., J. Bot. (Hooker) 4(31): 342. 1841. Type. Brazil. Piauí, “Serra de Araripe, near Caldas, Prov. Piauhy”, 1838–1841, *Gardner 1945* (lectotype: BM 000884631!, designated by Scalon et al. 2022; isolectotypes: E!, F!, G!, K!, NY!, OXF!, P!, W!).
Stryphnodendron obovatum Benth., Trans. Linn. Soc. London 30(3): 374. 1875. Type. Brazil. “Habitat inter Natividade et Porto Imperial, provinciae Goyaz”, May 1865, *Burchell 8343* (lectotype: K 000504730!, designated by Scalon et al. 2022; isolectotypes: F!, P!).
Stryphnodendron rotundifolium f. retusa Chodat & Hassl., Bull. Herb. Boissier, sér. 2, 4(6): 559. 1904. Type. Paraguay. “In campis cerrados in regione cursus superioris fluminis Apa”, Nov 1901–1902, *Hassler 7829* (lectotype: G 00400140!, designated by Scalon et al. 2022; isolectotypes: A, F!, G 00400103!, G 00400106!, G 00400108!, K!, MPU, NY!, P!, W!).

#### ﻿4.26.2 Stryphnodendron rotundifolium var. villosum

Taxon classificationPlantaeFabalesFabaceae

(Benth.) Scalon, Phytotaxa 544(3): 269. 2022.

9A38071D-3822-59FF-8CE6-ED8E1787EF68

Stryphnodendron goyazense Taub., Bot. Jahrb. Syst. 21(4): 434. 1896. Type. Brazil. “Habitat in locis Cerrados dictis prope Meiaponte”, Oct 1892, *Ule 2836* (lectotype: HBG 506635!, designated by Borges et al. 2018; isolectotype: P! [2], R!).
Stryphnodendron humile E.M.O. Martins, Leandra 6–7(7): 19. 1977. Type. Brazil. Minas Gerais, João Pinheiro, via Brasília-Minas, 30 Nov 1960, *Heringer 7783* (holotype: RFA 18438!; isotype: IAN!).

##### Basionym.

Stryphnodendronpolyphyllumvar.villosum Benth., Fl. Bras. 15(2): 285. 1876.

##### Type.

Brazil. “Prov. Sao Paulo”, s.d., *Burchell 5600* (lectotype: K 000504733!, designated by Scalon et al. 2022; isolectotypes: GH, P!).

#### ﻿4.27 Stryphnodendron velutinum

Taxon classificationPlantaeFabalesFabaceae

Scalon, Phytotaxa 544(3): 269. 2022.

9EF9C4EE-0B2A-5B2D-BD7A-DD3399D2A932

##### Type.

Brazil. Minas Gerais, Unaí, fragmento de cerradão no km 11 da rodovia Unaí/Paracatú, elev. 650 m, 16°15'S, 46°45'W, 22 Oct 1995, *Pereira & Alvarenga 2943* (holotype: IBGE 36575!; isotypes: CEN!, NY!, RB!, RFA!).

#### ﻿4.28 Stryphnodendron venosum

Taxon classificationPlantaeFabalesFabaceae

Scalon, Phytotaxa 544(3): 272. 2022.

AE8232A2-6BE9-5F55-836D-18E7BC6EED72

##### Type.

Bolivia. Santa Cruz: Ichilo, Reserva Florestal Choré, Rio Ibabo, Bosque Experimental “Elias Meneces”, 180 m, 16°35'S, 64°31'W, 16–18 Aug 1990, fr., *D. Neill & R. Quevedo 9361* (holotype: MO 3807891!; isotypes: G!, NY!, U!).

## Supplementary Material

XML Treatment for Microlobius

XML Treatment for Microlobius foetidus

XML Treatment for Microlobius foetidus subsp. paraguensis

XML Treatment for Gwilymia

XML Treatment for Gwilymia coriacea

XML Treatment for Gwilymia fissurata

XML Treatment for Gwilymia moricolor

XML Treatment for Gwilymia occhioniana

XML Treatment for Gwilymia paniculata

XML Treatment for Gwilymia polystachya

XML Treatment for Gwilymia racemifera

XML Treatment for Naiadendron

XML Treatment for Naiadendron duckeanum

XML Treatment for Stryphnodendron

XML Treatment for Stryphnodendron adstringens

XML Treatment for Stryphnodendron barbatulum

XML Treatment for Stryphnodendron confertum

XML Treatment for Stryphnodendron conicum

XML Treatment for Stryphnodendron cristalinae

XML Treatment for Stryphnodendron dryaticum

XML Treatment for Stryphnodendron excelsum

XML Treatment for Stryphnodendron flavotomentosum

XML Treatment for Stryphnodendron foreroi

XML Treatment for Stryphnodendron glandulosum

XML Treatment for Stryphnodendron gracile

XML Treatment for Stryphnodendron guianense

XML Treatment for Stryphnodendron heringeri

XML Treatment for Stryphnodendron holosericeum

XML Treatment for Stryphnodendron levelii

XML Treatment for Stryphnodendron microstachyum

XML Treatment for Stryphnodendron orinocense

XML Treatment for Stryphnodendron platycarpum

XML Treatment for Stryphnodendron platyspicum

XML Treatment for Stryphnodendron polyphyllum

XML Treatment for Stryphnodendron porcatum

XML Treatment for Stryphnodendron procerum

XML Treatment for Stryphnodendron pulcherrimum

XML Treatment for Stryphnodendron riparium

XML Treatment for Stryphnodendron roseiflorum

XML Treatment for Stryphnodendron rotundifolium

XML Treatment for Stryphnodendron rotundifolium Mart. var. rotundifolium

XML Treatment for Stryphnodendron rotundifolium var. villosum

XML Treatment for Stryphnodendron velutinum

XML Treatment for Stryphnodendron venosum

## ﻿Appendix 1

Voucher information for sequence data used in the phylogenetic analyses, all of which come from Koenen et al. (2020), Ringelberg et al. (2022) and Simon et al. (2016).

## Supplementary materials

Supplementary material 1 Figures S1–S18 Data type Phylogenetic Explanation note Figure S1. Phylogeny of mimosoid legumes based on combined transcriptome and hybrid capture data. Figures S2–S18. Optimization of characters 1–17 of Simon et al. (2016) over the 50% majority-rule consensus tree obtained in Bayesian analysis of molecular data. https://binary.pensoft.net/file/732750This dataset is made available under the Open Database License (http://opendatacommons.org/licenses/odbl/1.0/). The Open Database License (ODbL) is a license agreement intended to allow users to freely share, modify, and use this Dataset while maintaining this same freedom for others, provided that the original source and author(s) are credited.Alexandre Gibau de Lima, Juliana de Paula-Souza, Jens Ringelberg, Marcelo Fragomeni Simon, Luciano Paganucci de Queiroz, Leonardo M. Borges, Vidal de Freitas Mansano, Vinicius Castro Souza, Viviane Renata Scalon

Supplementary material 2 Table S1 Data type Phylogenetic Explanation note List of taxa and voucher information used in the phylogenomic analyses. https://binary.pensoft.net/file/732751This dataset is made available under the Open Database License (http://opendatacommons.org/licenses/odbl/1.0/). The Open Database License (ODbL) is a license agreement intended to allow users to freely share, modify, and use this Dataset while maintaining this same freedom for others, provided that the original source and author(s) are credited.Alexandre Gibau de Lima, Juliana de Paula-Souza, Jens Ringelberg, Marcelo Fragomeni Simon, Luciano Paganucci de Queiroz, Leonardo M. Borges, Vidal de Freitas Mansano, Vinicius Castro Souza, Viviane Renata Scalon

## References

[B1] Barroso GM Morim MP Peixoto AL Ichaso CLF (1999) Frutos e sementes: morfologia aplicada à sistemática de dicotiledôneas. UFV, Viçosa, 443 pp.

[B2] Bentham G (1841) Notes on Mimoseae, with a short synopsis of species. Le Journal de Botanique 4(31): 323–418.

[B3] Bentham G (1875) Revision of the suborder Mimoseae. Transactions of the Linnean Society of London 30(3): 335–375. 10.1111/j.1096-3642.1875.tb00005.x

[B4] Bentham G (1876) Leguminosae II et III. Mimoseae. In: Martius CFP (Ed.) Flora Brasiliensis, v.15, pars 2. Lipsiae apud Frid. Fleishcer in Comm., Monachii, 257–502.

[B5] Bolger AM Lohse M Usade B (2014) Trimmomatic: A flexible trimmer for Illumina sequence data. Bioinformatics 30(15): 2114–2120. 10.1093/bioinformatics/btu170 24695404 PMC4103590

[B6] Borges LM Schultz M Poppendieck H Kallunki JA Trovó M (2018) A tale of traded specimens, or what to know when selecting types from Ernst Ule’s collections. Taxon 67(3): 603. 10.12705/673.10

[B7] Borges LM Inglis PW Simon MF Ribeiro PG de Queiroz LP (2022) Misleading fruits: The non-monophyly of *Pseudopiptadenia* and *Pityrocarpa* supports generic re-circumscriptions and a new genus within mimosoid legumes. In: Hughes CE de Queiroz LP Lewis GP (Eds) Advances in Legume Systematics 14. Classification of Caesalpinioideae Part 1: New generic delimitations.PhytoKeys 205: 239–260. 10.3897/phytokeys.205.82275 PMC9849003 36762012

[B8] Brown JW Walker JF Smith SA (2017) Phyx: Phylogenetic tools for unix. Bioinformatics (Oxford, England) 33(12): 1886–1888. 10.1093/bioinformatics/btx063 28174903 PMC5870855

[B9] Caccavari MA (2002) Pollen morphology and structure of Tropical and Subtropical American genera of the Piptadenia-group (Leguminosae: Mimosoideae). Grana 41(3): 130–141. 10.1080/001731302321042597

[B10] Dexter KG Lavin M Torke BM Twyford AD Kursar TA Coley PD Drake C Hollands R Pennington T (2017) Dispersal assembly of rain forest tree communities across the Amazon basin. Proceedings of the National Academy of Sciences of the United States of America 114(10): 201613655. 10.1073/pnas.1613655114 PMC5347625 28213498

[B11] Guinet PH Caccavari MA (1992) Pollen morphology of the genus *Stryphnodendron* (Leguminosae, Mimosoideae) in relation to its taxonomy. Grana 31(2): 101–112. 10.1080/00173139209430729

[B12] Harris JG Harris MW (2001) Plant Identification Terminology: An Illustrated Glossary, 2^nd^ edn. Spring Lake Pub, Spring Lake, 216 pp.

[B13] Hu J-M Lavin M Wojciechowski MF Sanderson MJ (2000) Phylogenetic systematics of the tribe Millettieae (Leguminosae) based on chloroplast trnK/matK sequences and its implications for evolutionary patterns in the Papilionoideae. American Journal of Botany 87(3): 418–430. 10.2307/2656638 10719003

[B14] Hughes CE Ringelberg JJ Lewis GP Catalano SA (2022) Disintegration of the genus *Prosopis* L. (Leguminosae, Caesalpinioideae, mimosoid clade). In: Hughes CE de Queiroz LP Lewis GP (Eds) Advances in Legume Systematics 14. Classification of Caesalpinioideae Part 1: New generic delimitations.PhytoKeys 205: 147–190. 10.3897/phytokeys.205.75379 PMC9849005 36762004

[B15] Johnson MG Gardner EM Liu Y Medina R Goffinet B Shaw AJ Zerega NJC Wickett NJ (2016) HybPiper: Extracting coding sequence and introns for phylogenetics from high-throughput sequencing reads using target enrichment. Applications in Plant Sciences 4(7): e1600016. 10.3732/apps.1600016 PMC4948903 27437175

[B16] Koenen EJM Kidner CA de Souza ÉR Simon MF Iganci JRV Nicholls JA Brown GK de Queiroz LP Luckow MA Lewis GP Pennington RT Hughes CE (2020) Hybrid capture of 964 nuclear genes resolves evolutionary relationships in the mimosoid legumes and reveals the polytomous origins of a large pantropical radiation. American Journal of Botany 107(12): 1710–1735. 10.1002/ajb2.1568 33253423 PMC7839790

[B17] Lewis GP Elias TS (1981) Mimoseae. In: Polhill RM Raven PH (Eds) Advances in Legume Systematics.Pt 1. Royal Botanic Gardens, Kew, 155–169.

[B18] Lima AG Souza VC Paula-Souza J Scalon VR (2020) *Stryphnodendron* in Flora do Brasil 2020. Jardim Botânico do Rio de Janeiro. http://floradobrasil.jbrj.gov.br/reflora/floradobrasil/FB23174 [accessed 11.03. 2021].

[B19] Lima AG Paula-Souza J Scalon VR Souza VC (2021) *Stryphnodendronflavotomentosum* (Leguminosae, Caesalpinioideae, mimosoid clade), a new species from the Atlantic Forest, Brazil. Systematic Botany 46(1): 1–5. 10.1600/036364421X16128061189431

[B20] LPWG – The Legume Phylogeny Working Group (2017) A new subfamily classification of the Leguminosae based on a taxonomically comprehensive phylogeny. Taxon 66(1): 44–77. 10.12705/661.3

[B21] Maddison WP Maddison DR (2021) Mesquite: a modular system for evolutionary analysis. Version 3.70 http://www.mesquiteproject.org

[B22] Martius CFP (1837) Herbarium florae Brasiliensis. Munich [publisher not identified], 128 pp.

[B23] Miller MA Pfeiffer W Schwartz T (2010) Creating the CIPRES Science Gateway for inference of large phylogenetic trees. In Institute of Electrical and Electronics Engineers (Eds) Proceedings of the Gateway Computing Environments Workshop (GCE), November 14, 2010, New Orleans, LA, 1–8. 10.1109/GCE.2010.5676129

[B24] Möller M Cronk QCB (1997) Origin and relationships of *Saintpaulia* H. Wendl. (Gesneriaceae) based on ribosomal DNA internal transcribed spacer (ITS) sequences. American Journal of Botany 84(7): 956–965. 10.2307/2446286 21708650

[B25] Nicholls JA Pennington RT Koenen EJM Hughes CE Hearn J Bunnefeld L Dexter KG Stone GN Kidner CA (2015) Using targeted enrichment of nuclear genes to increase phylogenetic resolution in the neotropical rain forest genus *Inga* (Leguminosae: Mimosoideae). Frontiers in Plant Science 6: e710. 10.3389/fpls.2015.00710 PMC4584976 26442024

[B26] Occhioni P (1959) Duas espécies novas para a flora do Brasil. Revista Brasileira de Biologia 19(2): 207–209.

[B27] Occhioni EML (1990) Considerações taxonômicas no gênero *Stryphnodendron* Mart. (Leguminosae-Mimosoideae) e distribuição geográfica das espécies. Acta Botanica Brasílica 4(2): 153–158. 10.1590/S0102-33061990000300015

[B28] Occhioni-Martins EM (1981) *Stryphnodendron* Mart. (Leguminosae-Mimosoideae) com especial referência aos taxa amazônicos. Leandra 10–11: 3–100.

[B29] Paradis E Schliep K (2019) Ape 5.0: An environment for modern phylogenetics and evolutionary analyses in R. Bioinformatics 35(3): 526–528. 10.1093/bioinformatics/bty633 30016406

[B30] R Core Team (2022) R: A language and environment for statistical computing. R Foundation for Statistical Computing, Vienna. https://www.R-project.org/

[B31] Ranwez V Harispe S Delsuc F Douzery EJP (2011) MACSE: Multiple alignment of coding sequences accounting for frameshifts and stop codons. PLoS ONE 6(9): e22594. 10.1371/journal.pone.0022594 PMC3174933 21949676

[B32] Ribeiro PG Luckow M Lewis GP Simon MF Cardoso D Souza ER Silva APC Jesus MC Santos FAR Azevedo V Queiroz LP (2018) *Lachesiodendron*, a new monospecific genus segregated from *Piptadenia* (Leguminosae: Caesalpinioideae: mimosoid clade): Evidence from morphology and molecules. Taxon 67(1): 37–54. 10.12705/671.3

[B33] Ringelberg JJ Koenen EJM Iganci JR de Queiroz LP Murphy DJ Gaudeul M Bruneau A Luckow M Lewis GP Hughes CE (2022) Phylogenomic analysis of 997 nuclear genes reveals the need for extensive generic re-delimitation in Caesalpinioideae (Leguminosae). In: Hughes CE de Queiroz LP Lewis GP (Eds) Advances in Legume Systematics 14. Classification of Caesalpinioideae Part 1: New generic delimitations.PhytoKeys 205: 3–58. 10.3897/phytokeys.205.85866 PMC9848904 36762007

[B34] Ronquist F Teslenko M van der Mark P Ayres DL Darling A Höhna S Larget B Liu L Suchard MA Huelsenbeck JP (2012) MrBayes 3.2: Efficient Bayesian phylogenetic inference and model choice across a large model space. Systematic Biology 61(3): 539–542. 10.1093/sysbio/sys029 22357727 PMC3329765

[B35] Scalon VR (2007) Revisão taxonômica do gênero Stryphnodendron Mart. (Leguminosae-Mimosoideae). PhD thesis. Universidade de São Paulo.

[B36] Scalon VR Paula-Souza J Lima AG Souza VC (2022) A synopsis of the genus *Stryphnodendron* (Fabaceae, Caesalpinioideae, mimosoid clade). Phytotaxa 544(3): 227–279. 10.11646/phytotaxa.544.3.1

[B37] Shorthouse DP (2010) SimpleMappr, an online tool to produce publication-quality point maps. https://www.simplemappr.net

[B38] Simon MF Grether R Queiroz LP Skema C Pennington RT Hughes CE (2009) Recent assembly of the Cerrado, a Neotropical plant diversity hotspot, by in situ evolution of adaptations to fire. Proceedings of the National Academy of Sciences of the United States of America 106(48): 20359–20364. 10.1073/pnas.0903410106 19918050 PMC2787167

[B39] Simon MF Grether R Queiroz LP Särkinen TE Dutra VF Hughes CE (2011) The evolutionary history of *Mimosa* (Leguminosae): Toward a phylogeny of the sensitive plants. American Journal of Botany 98(7): 1201–1221. 10.3732/ajb.1000520 21730340

[B40] Simon MF Pastore JFB Souza AF Borges LM Scalon VR Ribeiro PG Santos-Silva J Souza VC Queiroz LP (2016) Molecular phylogeny of *Stryphnodendron* (Mimosoideae, Leguminosae) and generic delimitations in the *Piptadenia* Group. International Journal of Plant Sciences 177(1): 44–59. 10.1086/684077

[B41] Smith SA Moore MJ Brown JW Yang Y (2015) Analysis of phylogenomic datasets reveals conflict, concordance, and gene duplications with examples from animals and plants. BMC Evolutionary Biology 15(1): e150. 10.1186/s12862-015-0423-0 PMC4524127 26239519

[B42] Sousa MS Andrade GM (1992) Identidad de *Microlobius* y *Goldmania* (Leguminosae: Mimosoideae: Mimoseae) y nuevas combinaciones. Anales del Instituto de Biología de la Universidad Nacional Autónoma de México. Botánica 63(1): 101–107.

[B43] Souza ER Lewis GP Forest F Schnadelbach AS Van der Berg C Queiroz LP (2013) Phylogeny of *Calliandra* (Leguminosae: Mimosoideae) based on nuclear and plastid molecular markers. Taxon 62(6): 1200–1219. 10.12705/626.2

[B44] Stafleu FA Cowan RS (1976) Taxonomic Literature. A selective guide to botanical publications and collections with dates, commentaries and types. Vol. 1 A-G. 2^nd^ edn. Bohn, Scheltema & Holkema, Utrecht, 1136 pp. 10.5962/bhl.title.48631

[B45] Stafleu FA Cowan RS (1979) Taxonomic Literature. A selective guide to botanical publications and collections with dates, commentaries and types. Vol. 2 H-Le. 2^nd^ edn. Bohn, Scheltema & Holkema, Utrecht. Dr. W. Junk b.v., Publishers, The Hague, 991 pp. 10.5962/bhl.title.48631

[B46] Stamatakis A (2014) RAxML version 8: A tool for phylogenetic analysis and post-analysis of large phylogenies. Bioinformatics 30(9): 1312–1313. 10.1093/bioinformatics/btu033 24451623 PMC3998144

[B47] Swofford DL (2003) PAUP*. Phylogenetic Analysis Using Parsimony (*and Other Methods). Version 4. Sinauer Associates, Sunderland, Massachusetts.

[B48] Taberlet P Gielly L Patou G Bouvet J (1991) Universal primers for amplification of three non-coding regions of chloroplast DNA. Plant Molecular Biology 17(5): 1105–1109. 10.1007/BF00037152 1932684

[B49] Thiers B (Ed.) [2018] Index Herbariorum: a global directory of public herbaria and associated staff. New York Botanical Garden’s Virtual Herbarium. http://sweetgum.nybg.org/science/ih/ [accessed 15.05.2018]

[B50] Weberling F (1989) Morphology of flowers and inflorescences. Cambridge University Press, Cambridge, 405 pp.

[B51] White TJ Bruns T Lee S Taylor J (1990) Amplification and direct sequencing of fungal ribosomal RNA genes for phylogenetics. In: Innis M, Gelfand D, Sninsky J, White Y (Eds) PCR protocols: a guide to methods and applications. Academic Press, San Diego, CA. 315–322. 10.1016/B978-0-12-372180-8.50042-1

[B52] Wojciechowski MF Lavin M Sanderson M (2004) A phylogeny of legumes (Leguminosae) based on analysis of the plastid matK gene resolves many well-supported subclades within the family. American Journal of Botany 91(11): 1846–1862. 10.3732/ajb.91.11.1846 21652332

[B53] Yang Y Smith SA (2014) Orthology inference in nonmodel organisms using transcriptomes and low-coverage genomes: improving accuracy and matrix occupancy for phylogenomics. Molecular Biology and Evolution 31(11): 3081–3092. 10.1093/molbev/msu245 25158799 PMC4209138

[B54] Zhang C Scornavacca C Molloy EK Mirarab S (2020) ASTRAL-Pro: Quartet-based species-tree inference despite paralogy. Molecular Biology and Evolution 37(11): 3292–3307. 10.1093/molbev/msaa139 32886770 PMC7751180

