# Supplementary material for: ﻿New segregates from the Neotropical genus Stryphnodendron (Leguminosae, Caesalpinioideae, mimosoid clade)
Source: PhytoKeys. 2022 Aug 22;205:203–37. doi: 10.3897/phytokeys.205.82220 (PMC9849044; doi:10.3897/phytokeys.205.82220)
Supplement: Supplementary material 1 — Figures S1–S18 [file phytokeys-205-203_article-82220__-s001.pdf]

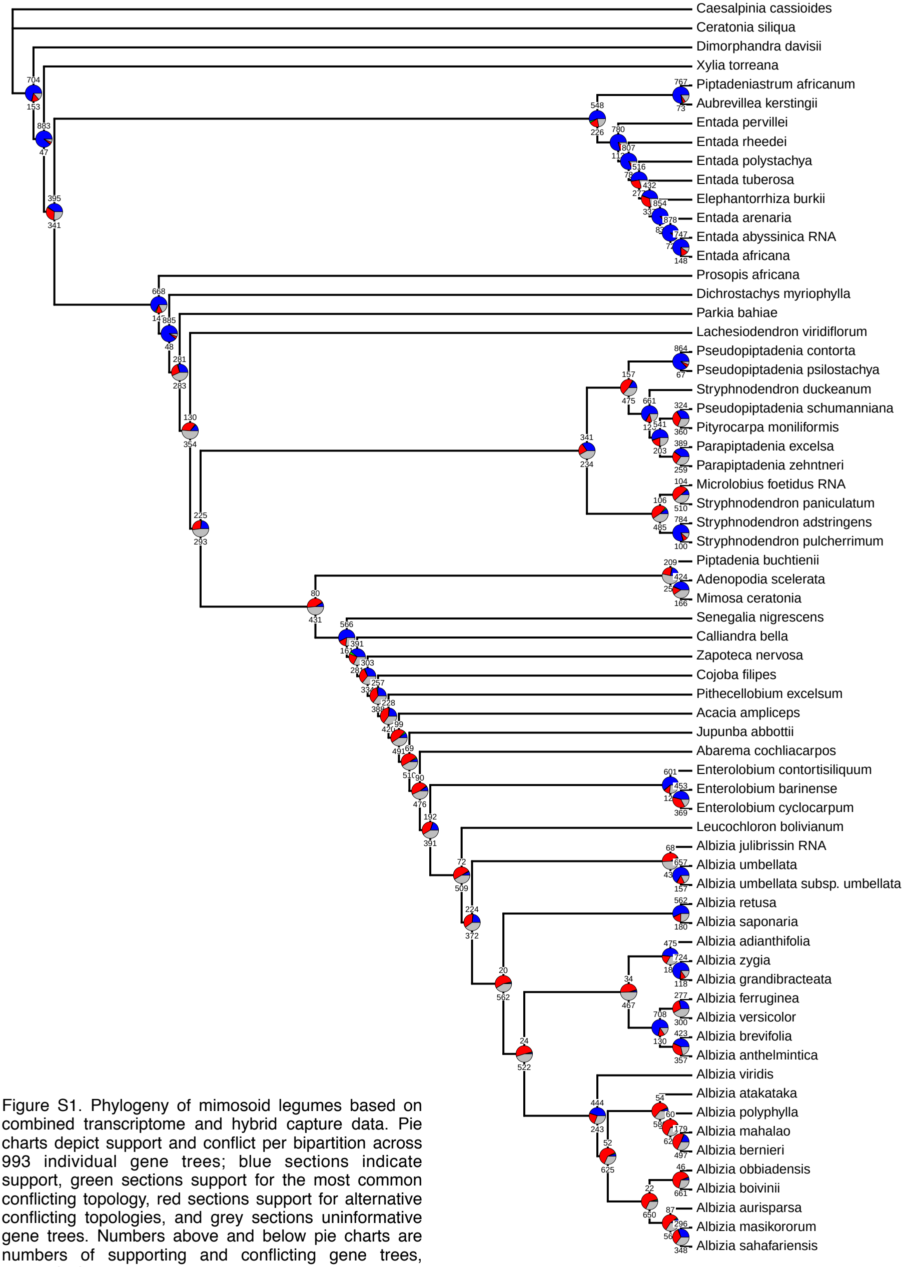

Figure S1. Phylogeny of mimosoid legumes based on combined transcriptome and hybrid capture data. Pie charts depict support and conflict per bipartition across 993 individual gene trees; blue sections indicate support, green sections support for the most common conflicting topology, red sections support for alternative conflicting topologies, and grey sections uninformative gene trees. Numbers above and below pie charts are numbers of supporting and conflicting gene trees, respectively.

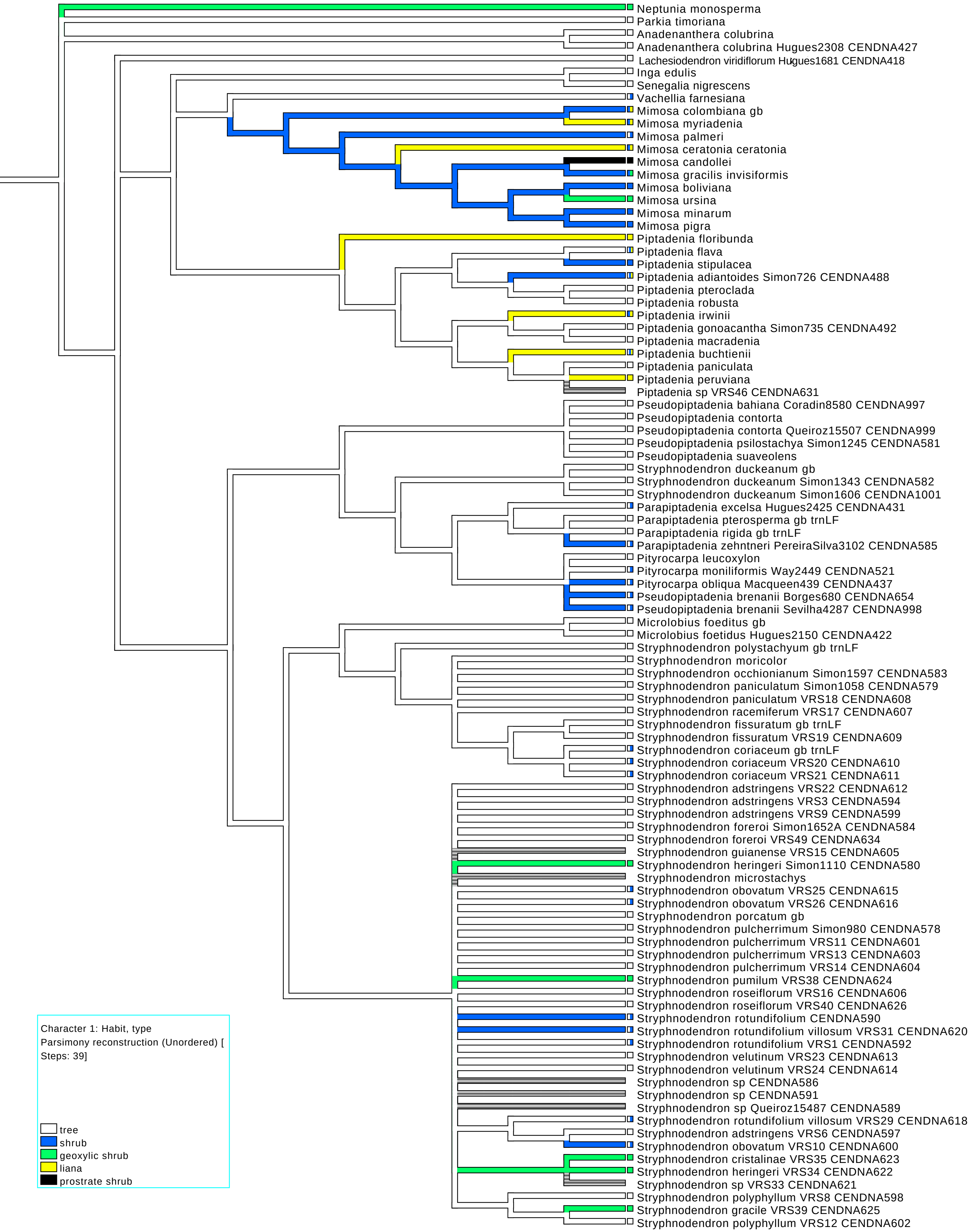

Figure S2. Optimization of character 1 of Simon et al. (2016) over the 50% majority-rule consensus tree obtained in Bayesian analysis of molecular data.

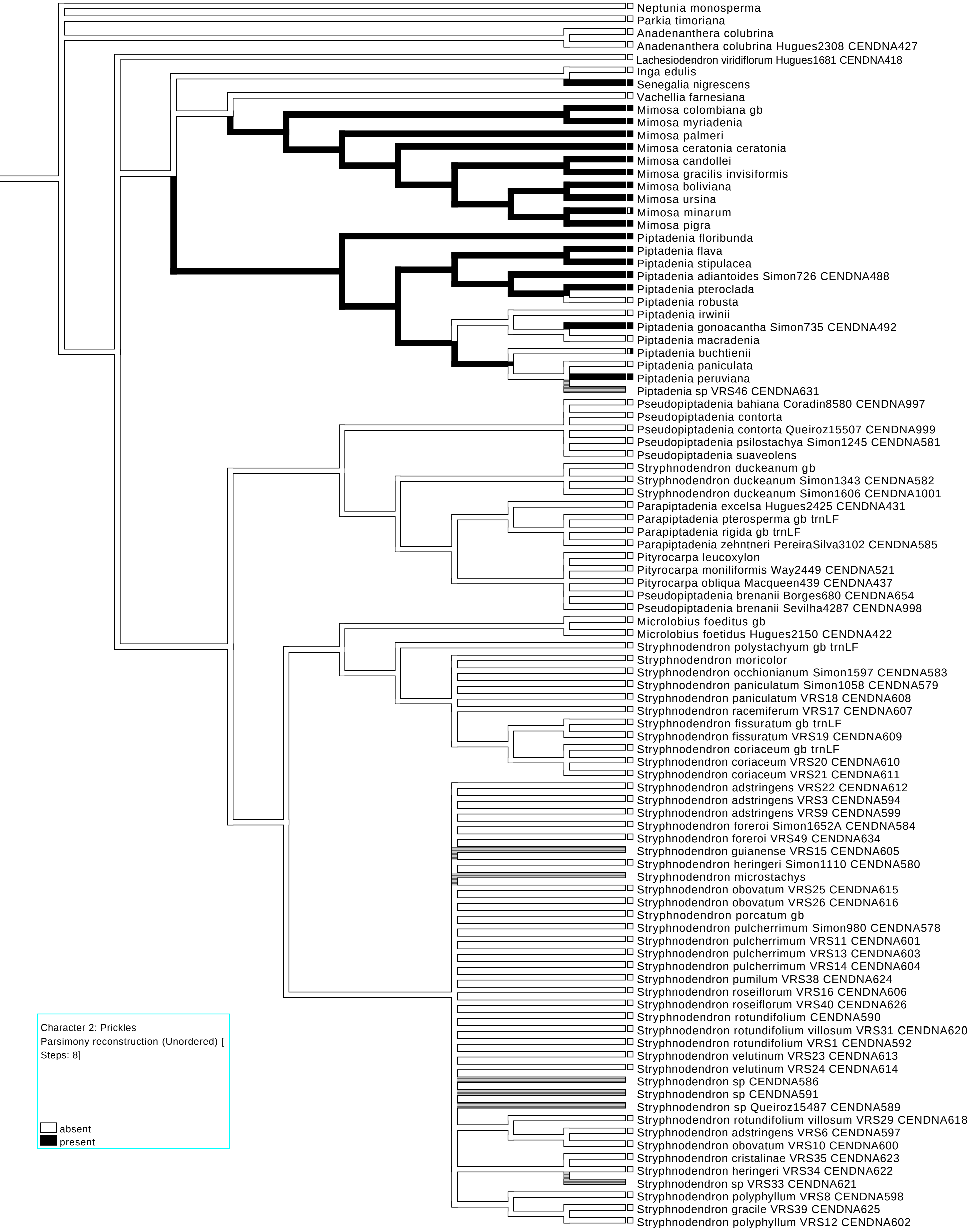

Figure S3. Optimization of character 2 of Simon et al. (2016) over the 50% majority-rule consensus tree obtained in Bayesian analysis of molecular data.

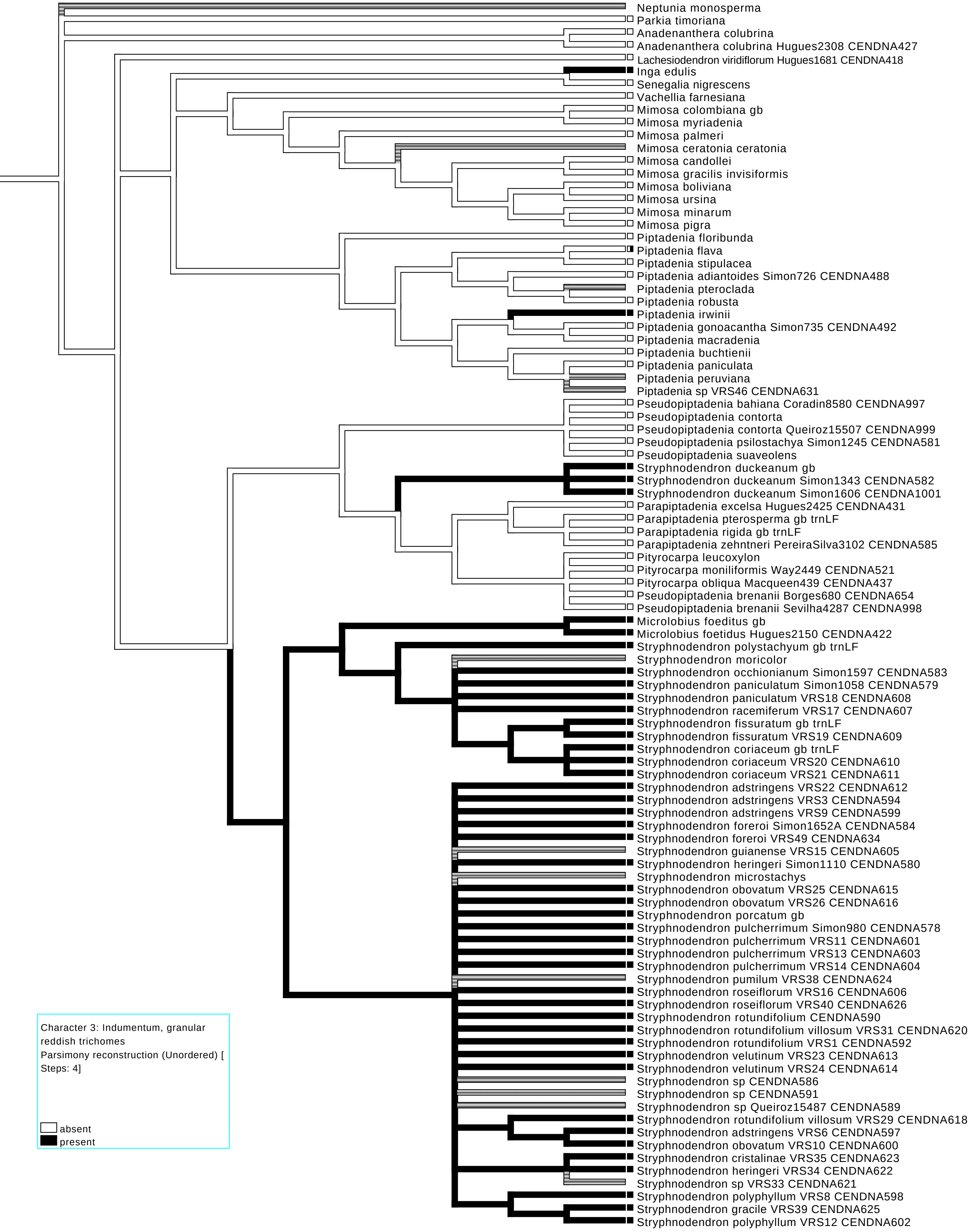

Figure S4. Optimization of character 3 of Simon et al. (2016) over the 50% majority-rule consensus tree obtained in Bayesian analysis of molecular data.

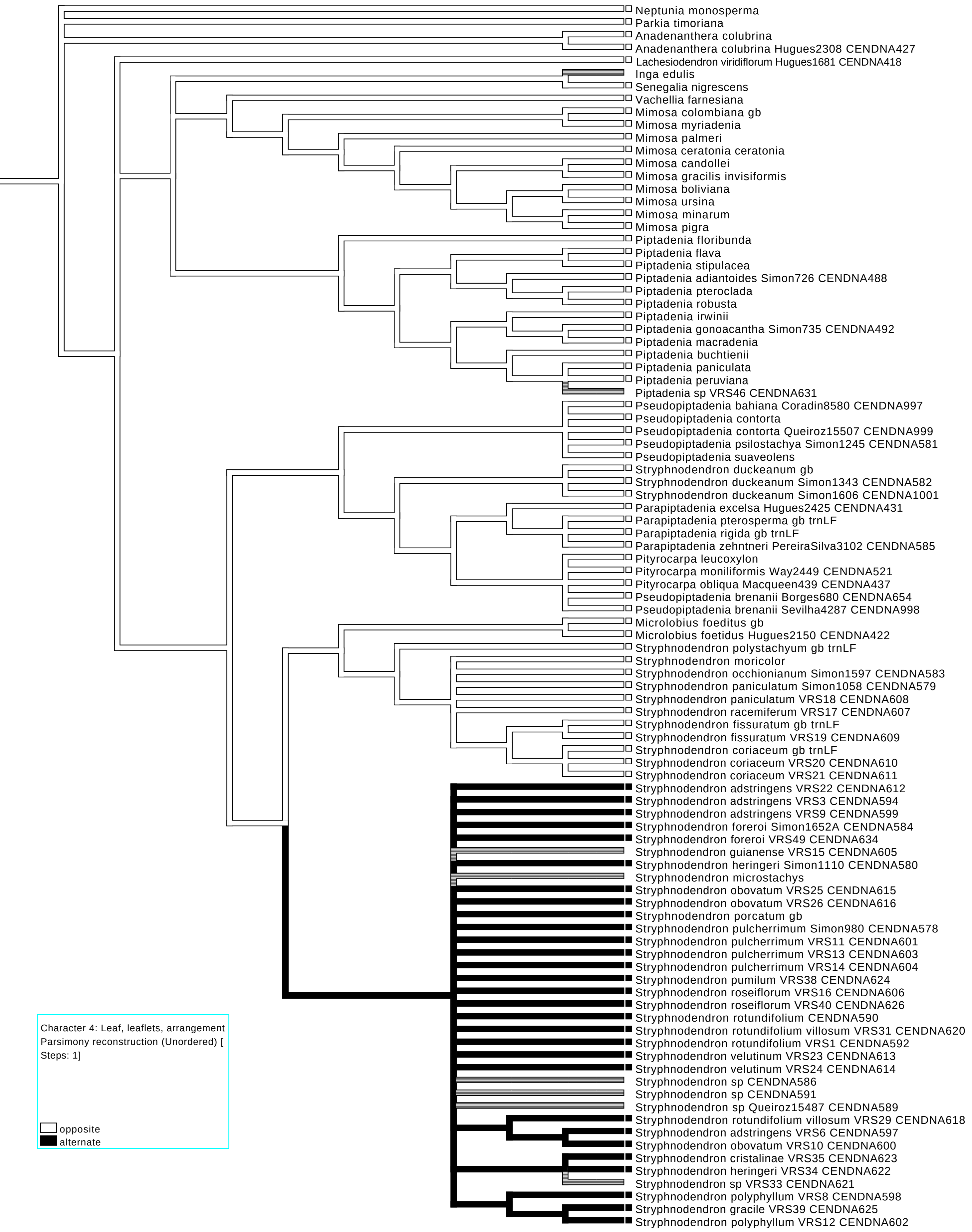

Figure S5. Optimization of character 4 of Simon et al. (2016) over the 50% majority-rule consensus tree obtained in Bayesian analysis of molecular data.

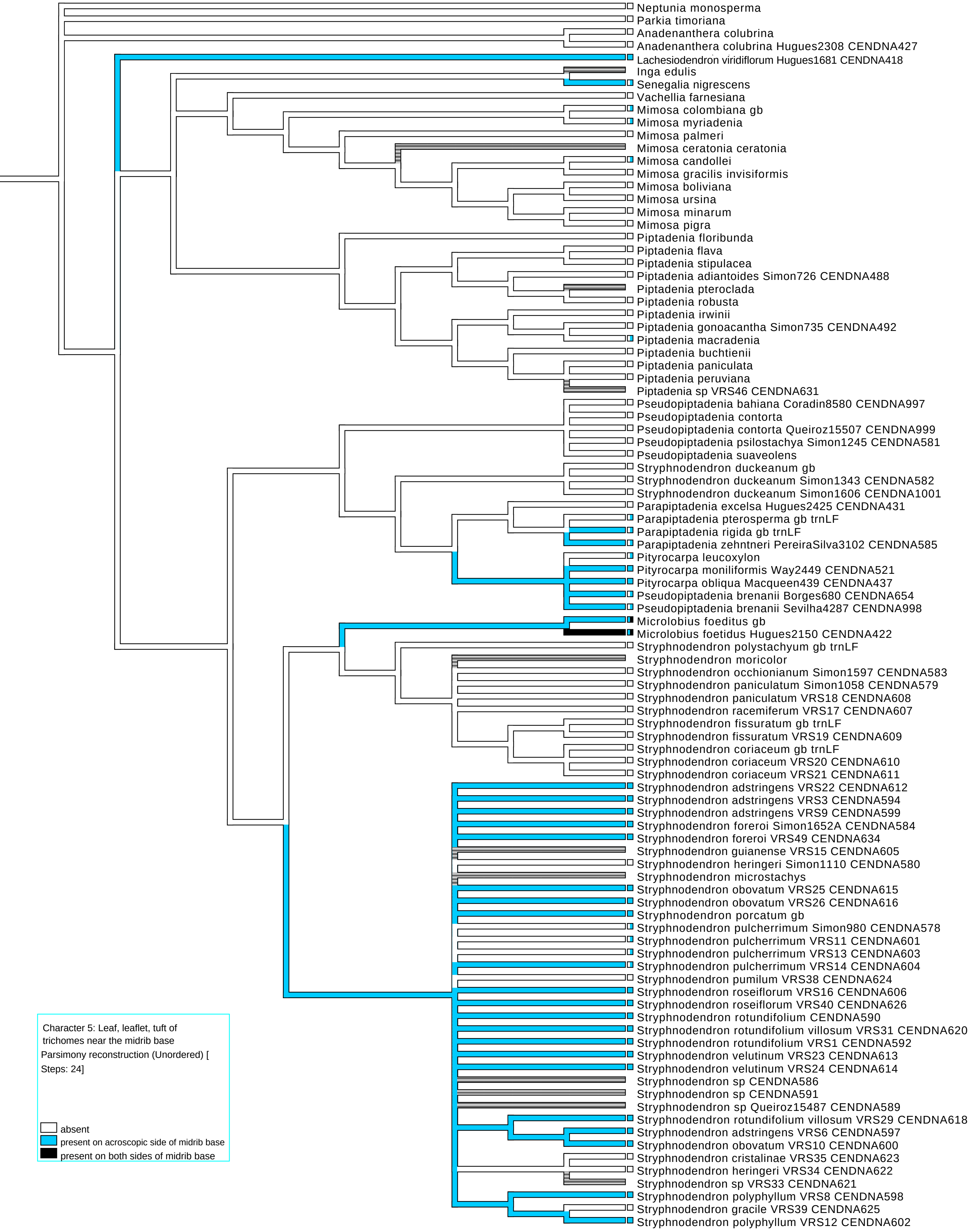

Figure S6. Optimization of character 5 of Simon et al. (2016) over the 50% majority-rule consensus tree obtained in Bayesian analysis of molecular data.

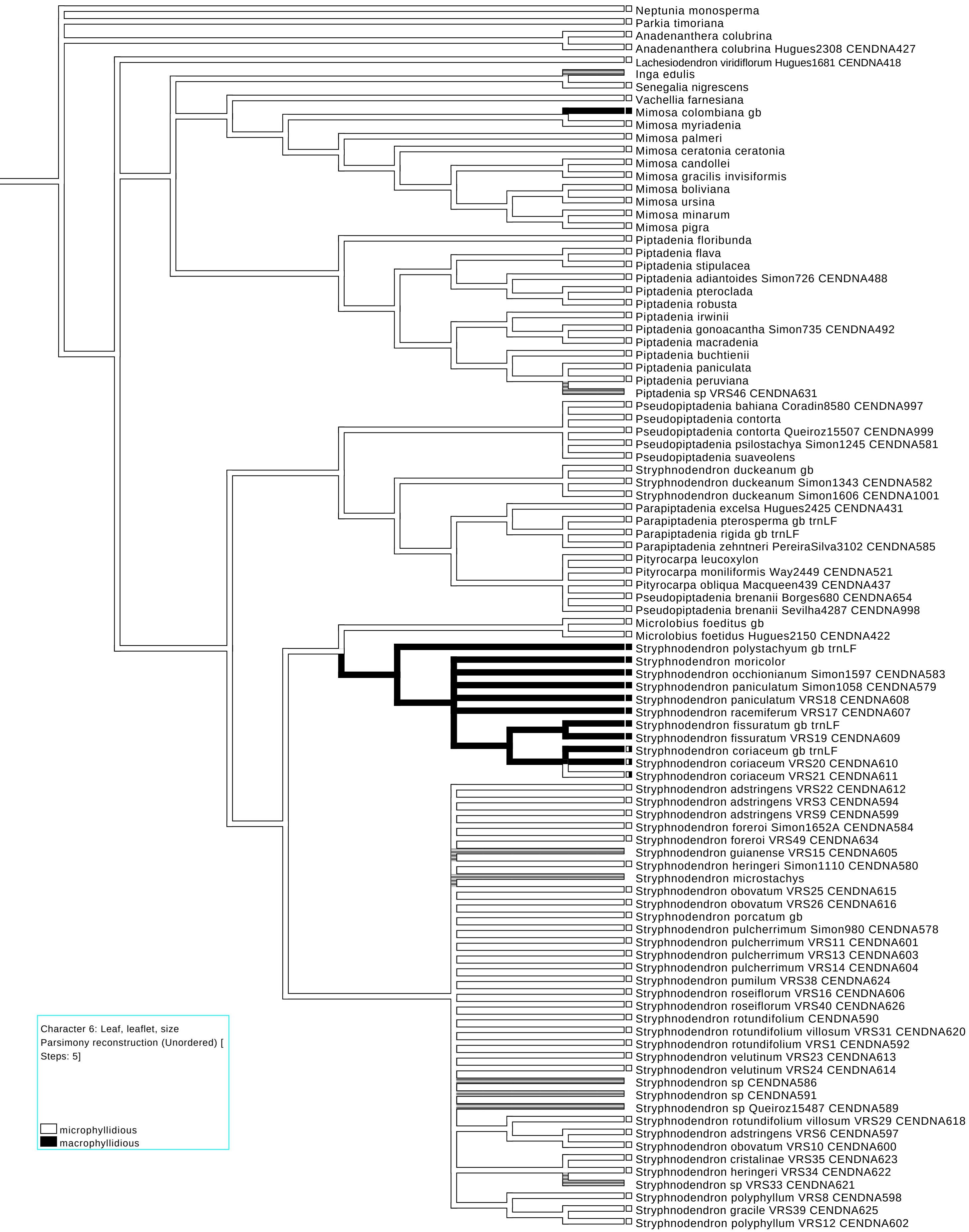

Figure S7. Optimization of character 6 of Simon et al. (2016) over the 50% majority-rule consensus tree obtained in Bayesian analysis of molecular data.

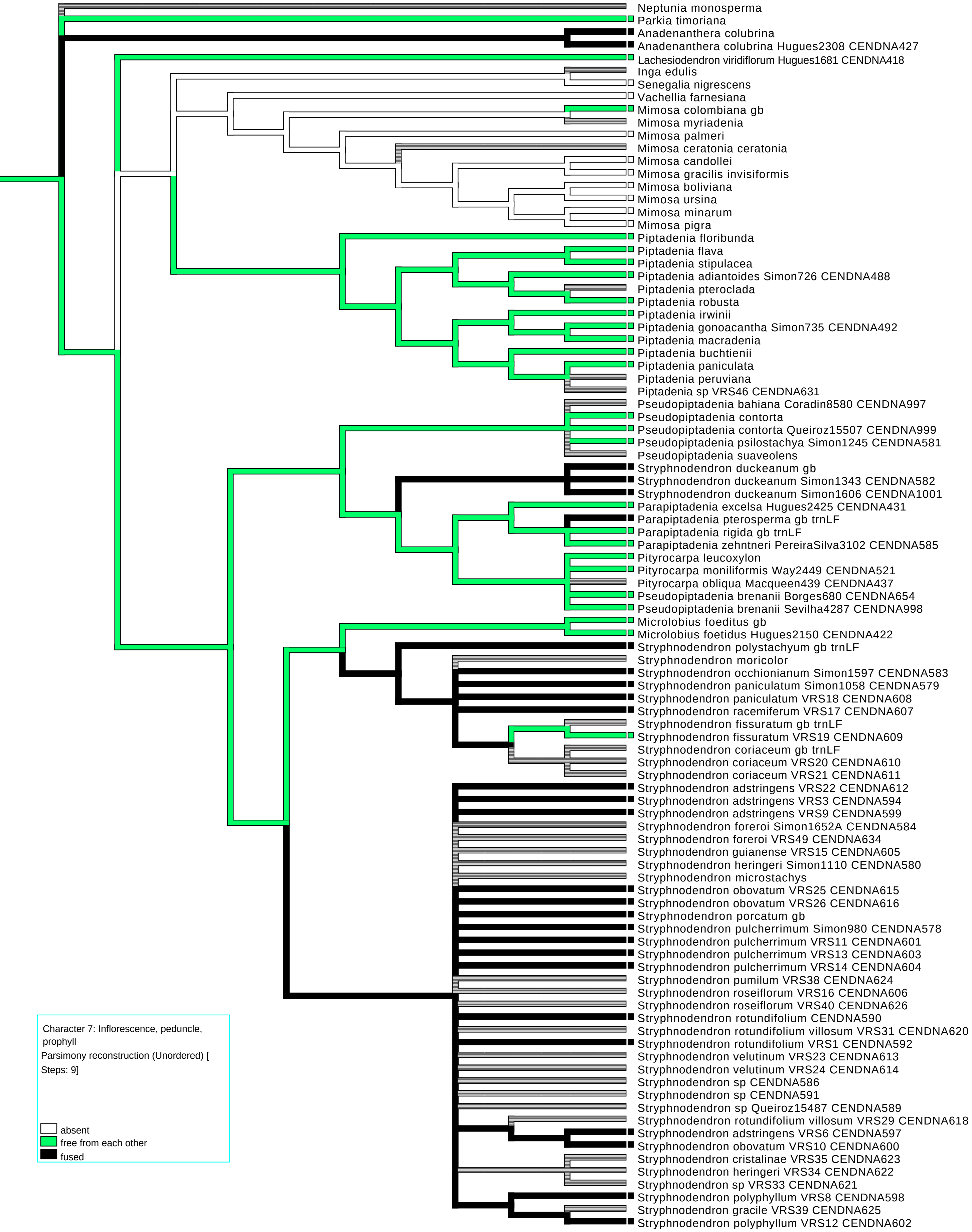

Figure S8. Optimization of character 7 of Simon et al. (2016) over the 50% majority-rule consensus tree obtained in Bayesian analysis of molecular data.

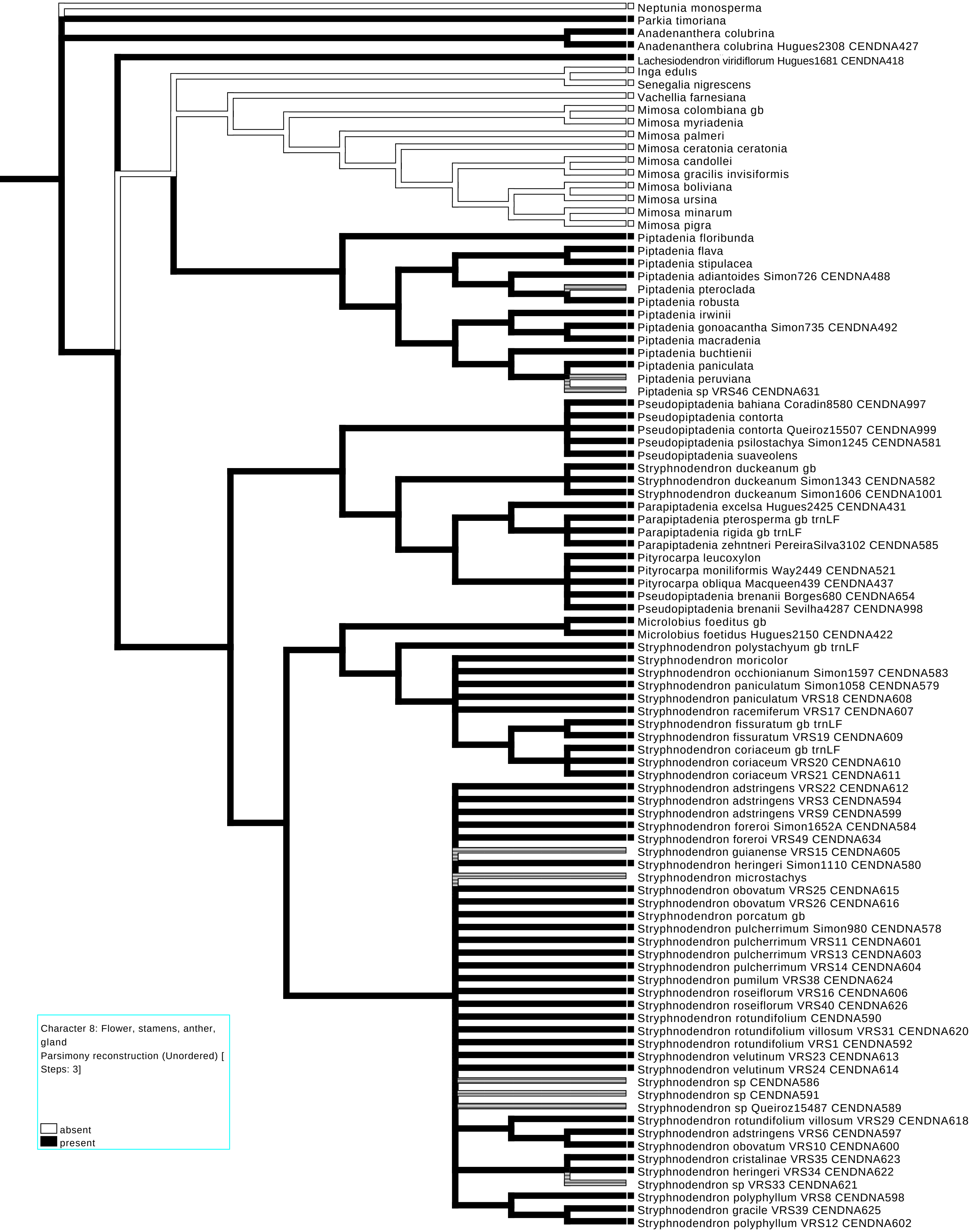

Figure S9. Optimization of character 8 of Simon et al. (2016) over the 50% majority-rule consensus tree obtained in Bayesian analysis of molecular data.

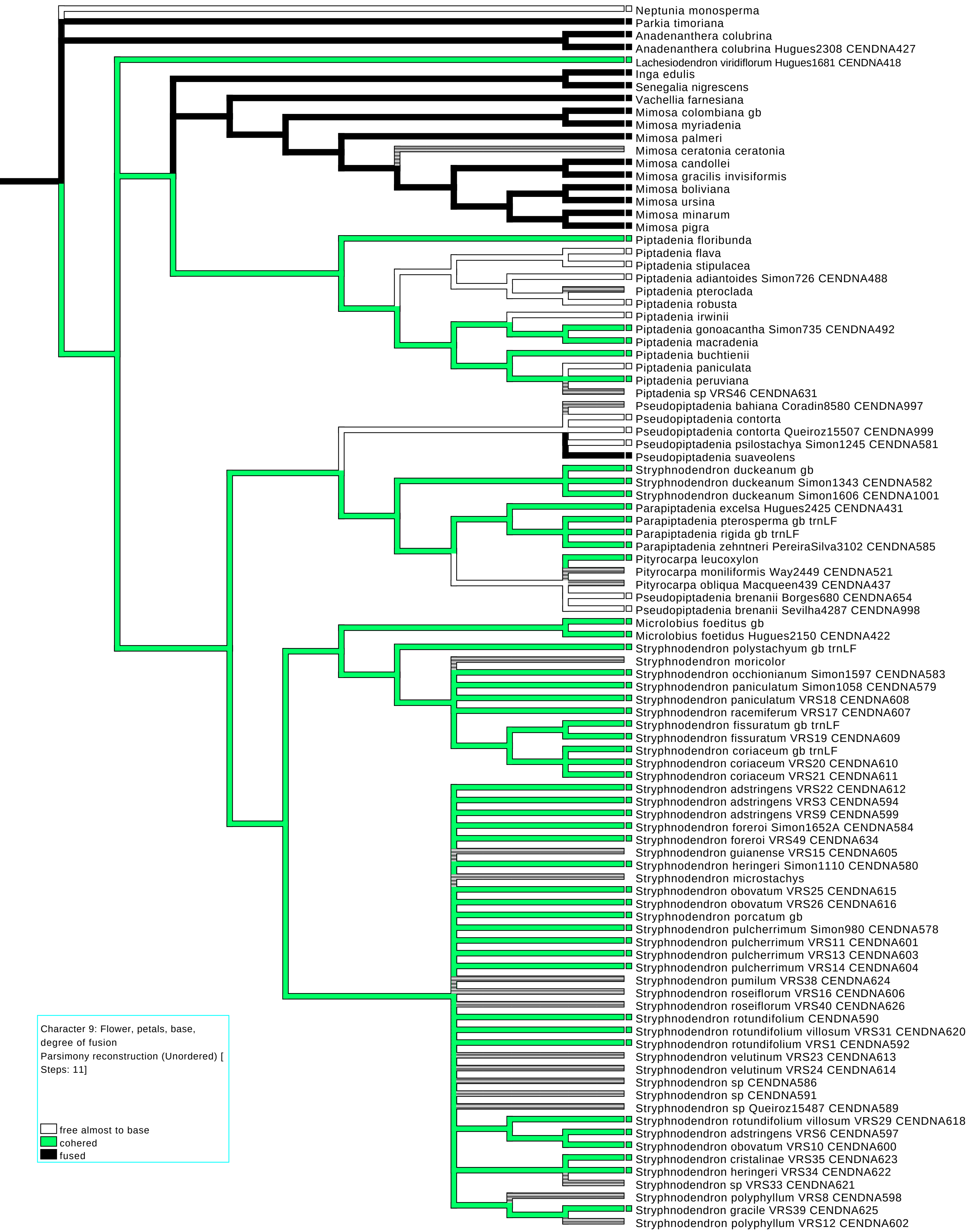

Figure S10. Optimization of character 9 of Simon et al. (2016) over the 50% majority-rule consensus tree obtained in Bayesian analysis of molecular data. "Cohered" refers to the attachment of the petals without epidermal fusion (suture line between the petals present). “ Fused” is applied when epidermal fusion is inferred (suture line between the petals absent), for more details see Simon et al. (2016).

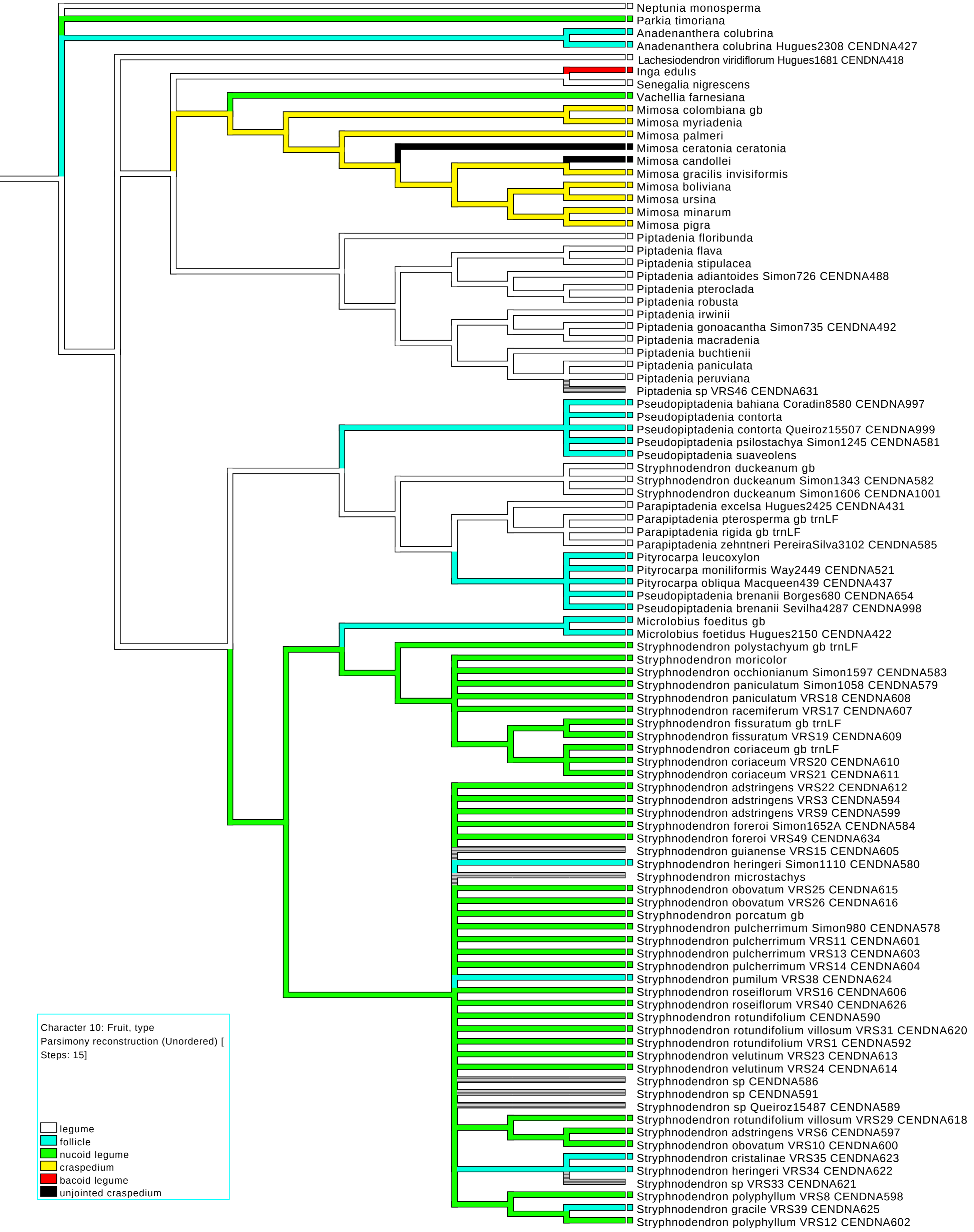

Figure S11. Optimization of character 10 of Simon et al. (2016) over the 50% majority-rule consensus tree obtained in Bayesian analysis of molecular data.

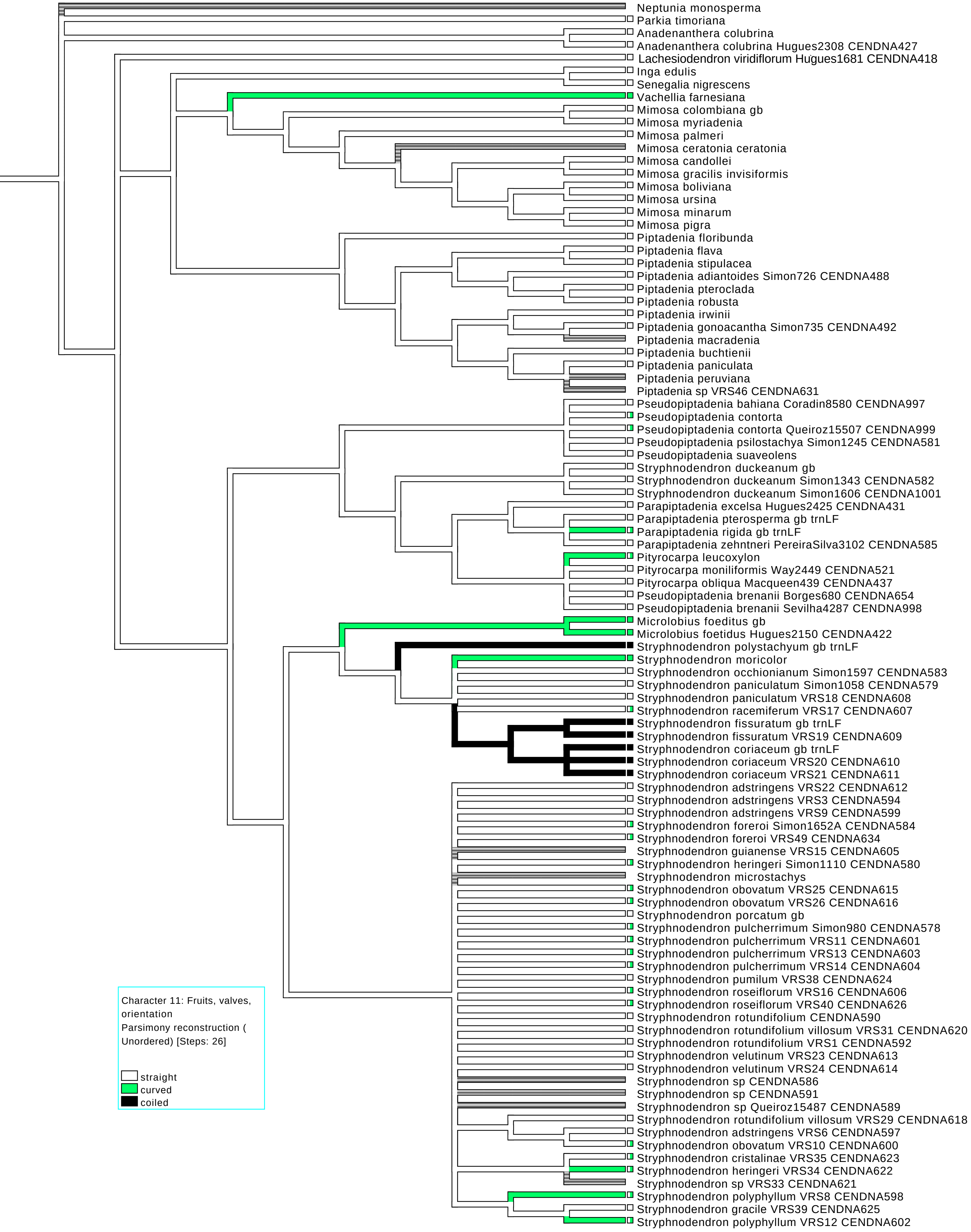

Figure S12. Optimization of character 11 of Simon et al. (2016) over the 50% majority-rule consensus tree obtained in Bayesian analysis of molecular data.

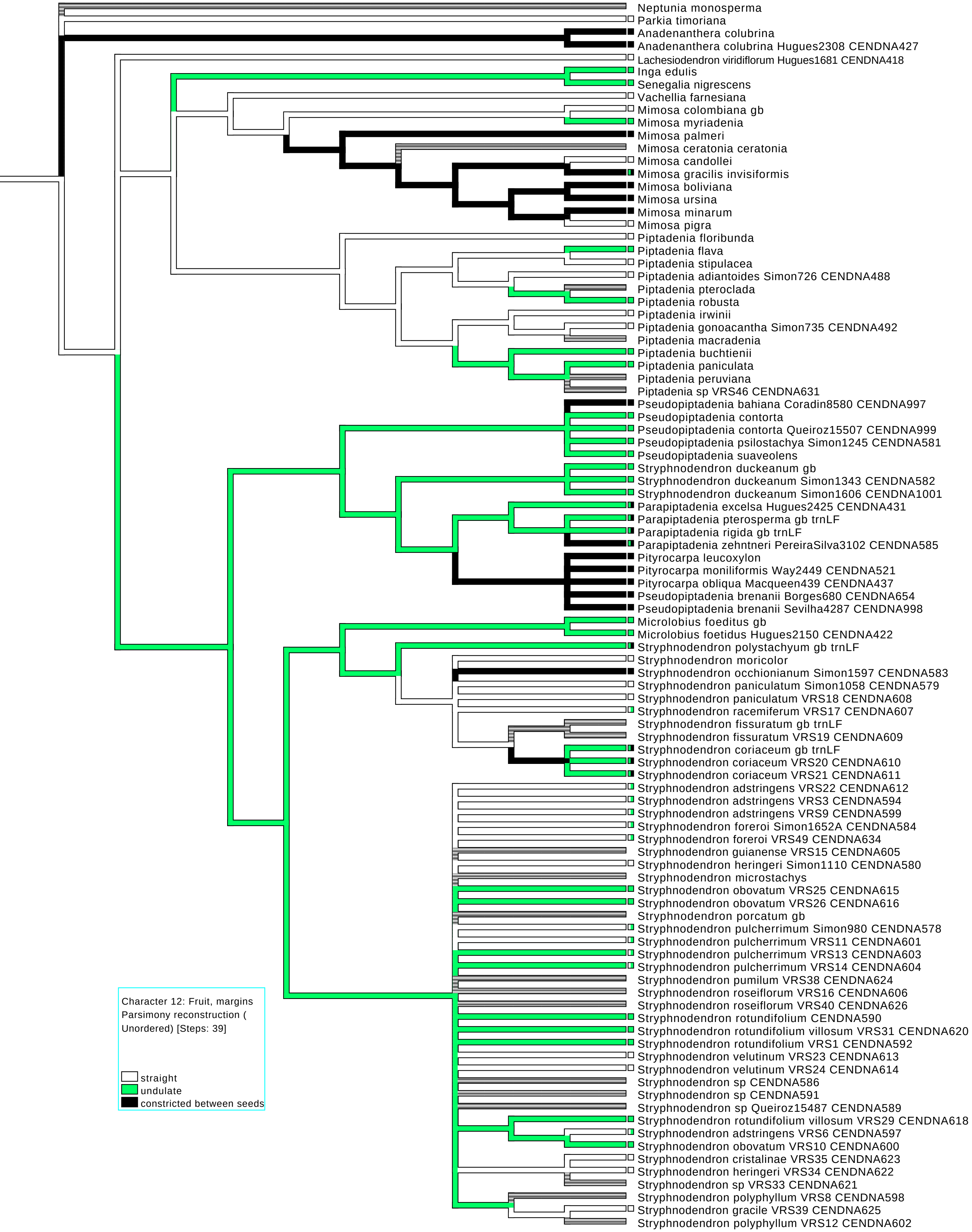

Figure S13. Optimization of character 12 of Simon et al. (2016) over the 50% majority-rule consensus tree obtained in Bayesian analysis of molecular data.

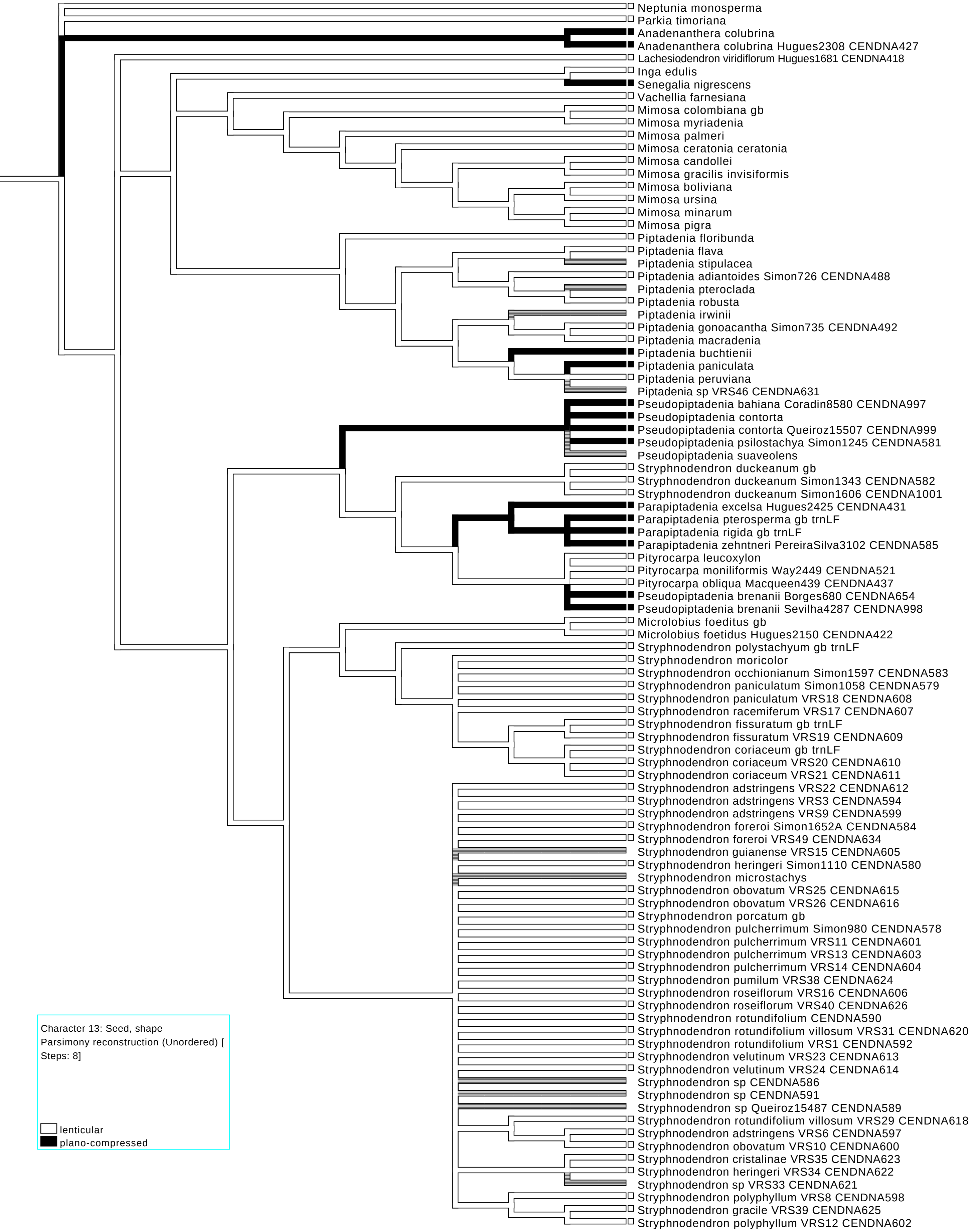

Figure S14. Optimization of character 13 of Simon et al. (2016) over the 50% majority-rule consensus tree obtained in Bayesian analysis of molecular data.

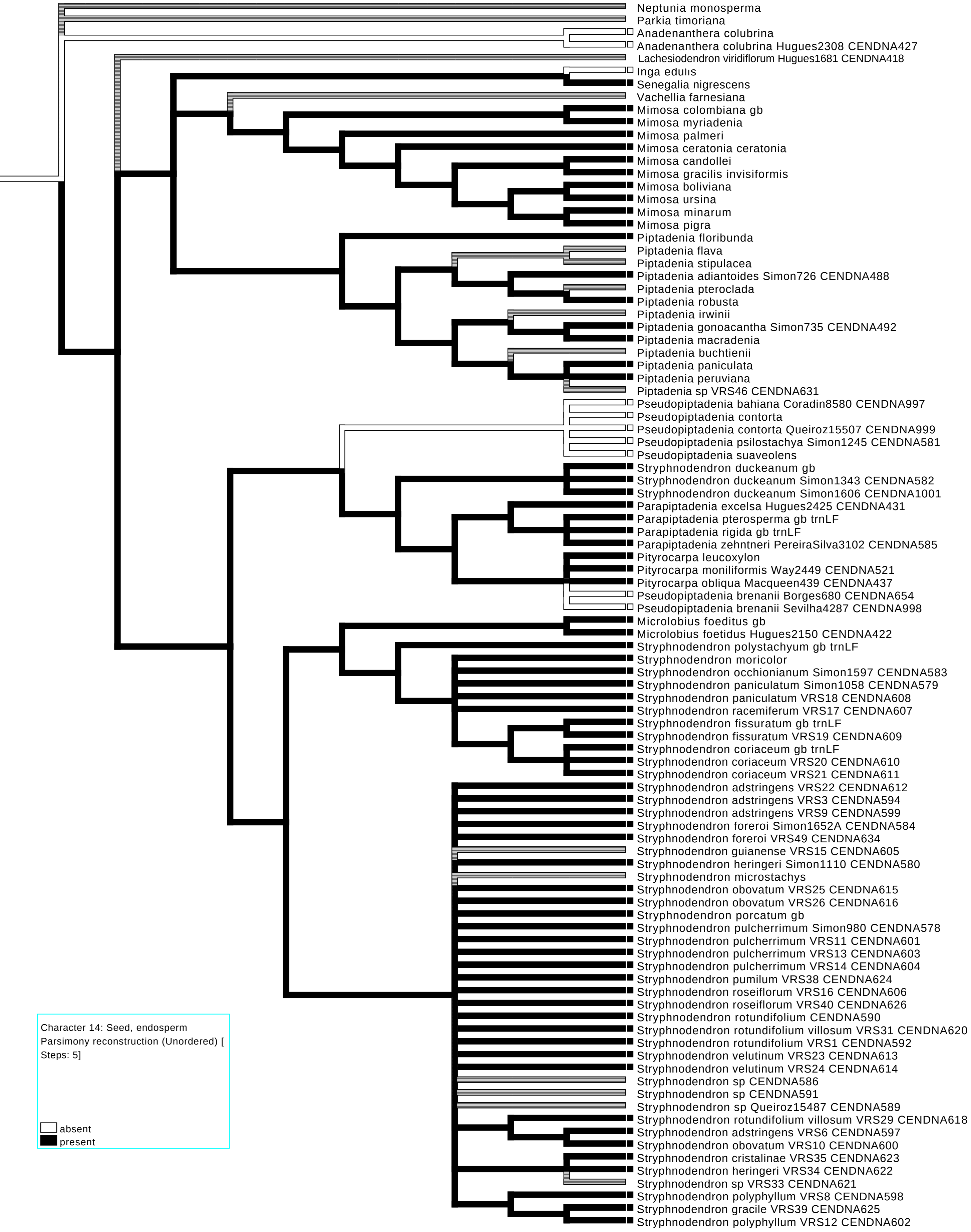

Figure S15. Optimization of character 14 of Simon et al. (2016) over the 50% majority-rule consensus tree obtained in Bayesian analysis of molecular data.

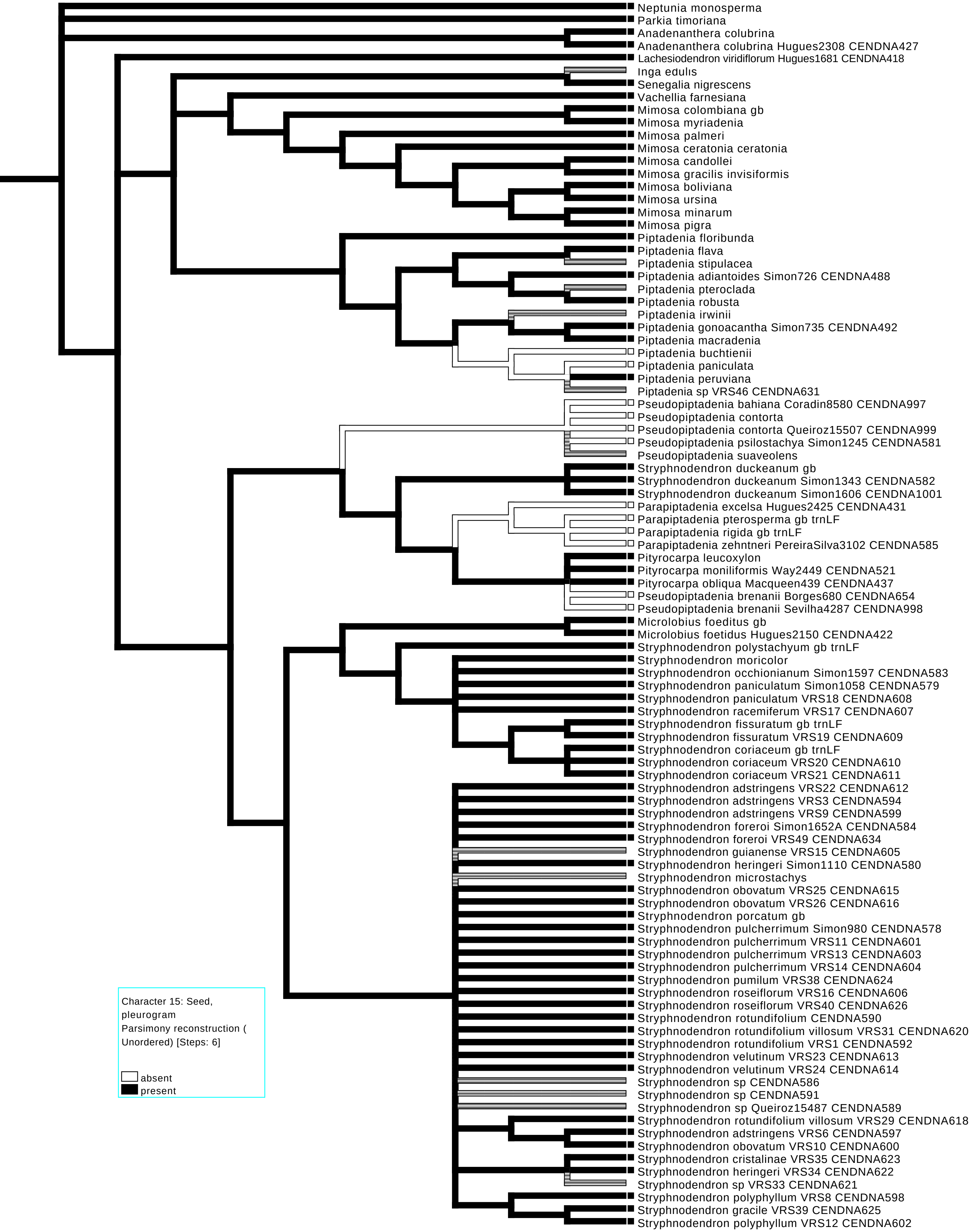

Figure S16. Optimization of character 15 of Simon et al. (2016) over the 50% majority-rule consensus tree obtained in Bayesian analysis of molecular data.

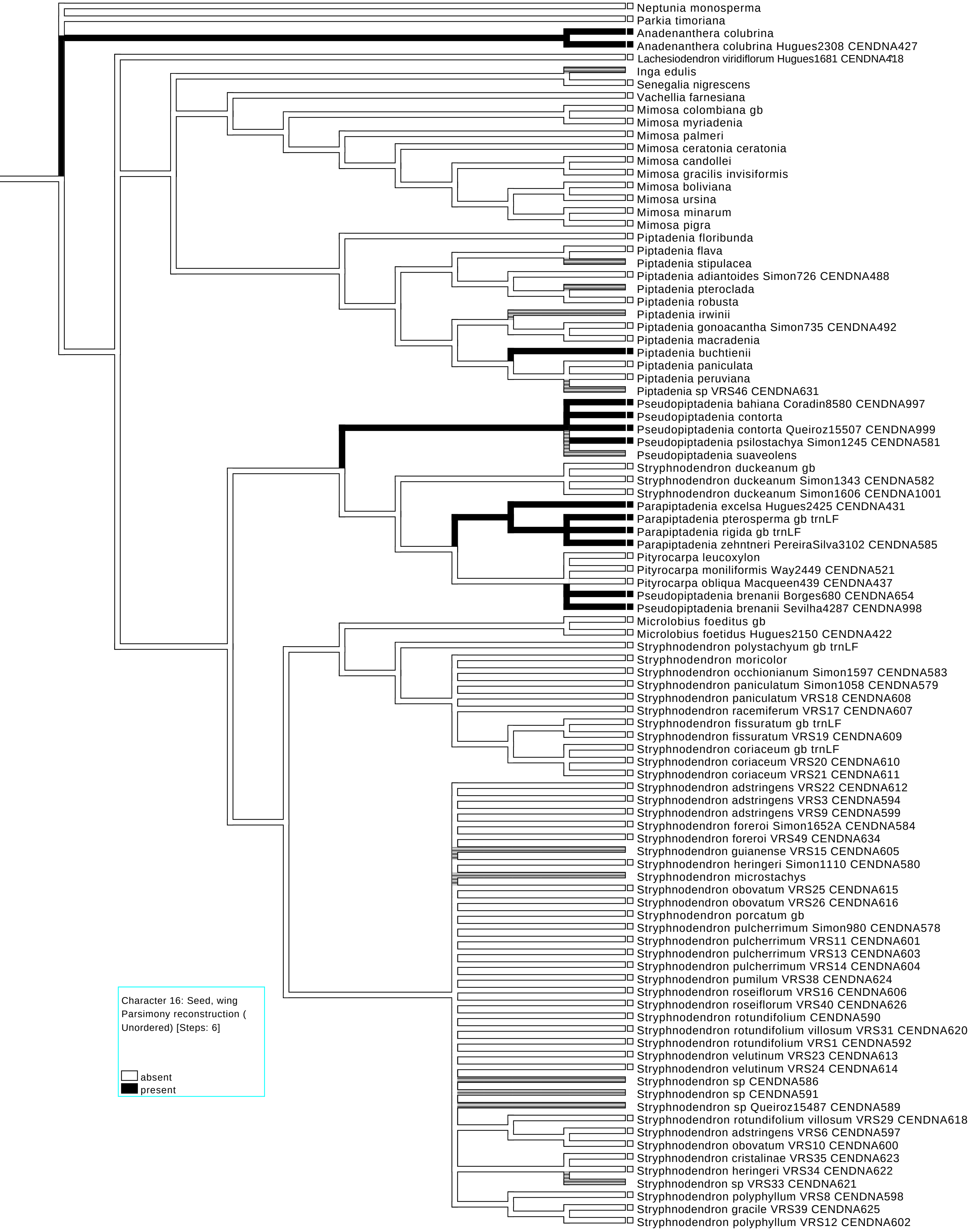

Figure S17. Optimization of character 16 of Simon et al. (2016) over the 50% majority-rule consensus tree obtained in Bayesian analysis of molecular data.

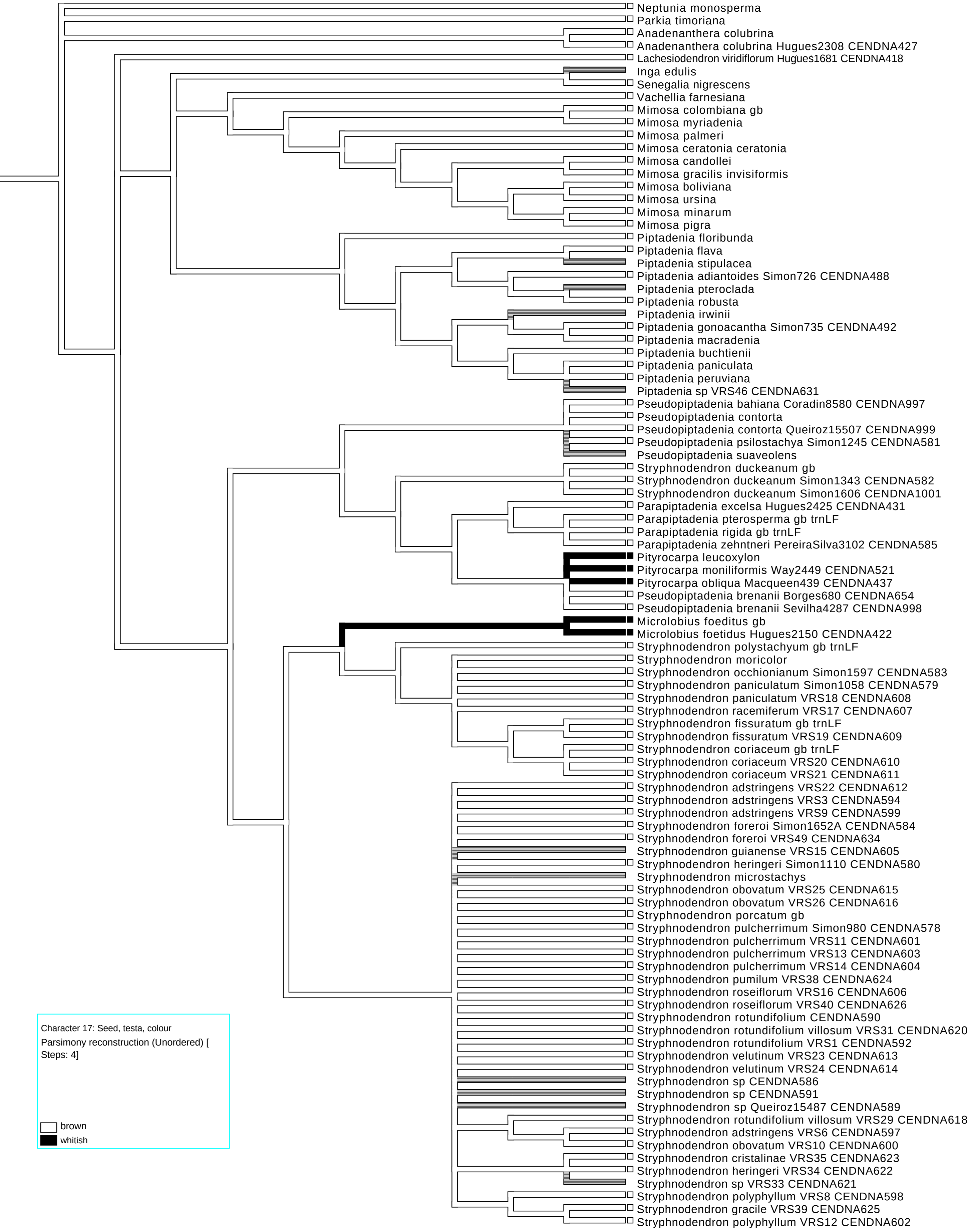

Figure S18. Optimization of character 17 of Simon et al. (2016) over the 50% majority-rule consensus tree obtained in Bayesian analysis of molecular data.
